# Improving vulnerable Calbindin1^−^ neurons in the ventral hippocampus rescues tau-induced impairment of episodic memory

**DOI:** 10.1186/s40035-025-00473-w

**Published:** 2025-03-04

**Authors:** Huiyang Lei, Jingru Lv, Fuqiang Zhang, Linyu Wei, Kun Shi, Jiale Liu, Ting He, Rui Xiong, Fei Sun, Tongkai Zhong, Jingqi Zhao, Dan Ke, Qun Wang, Peiran Jiang, Ai-Min Bao, Jian-Zhi Wang, Ying Yang

**Affiliations:** 1 Department of Pathophysiology, School of Basic Medicine, Key Laboratory of Education Ministry of China/Hubei Province for Neurological Disorders, Tongji Medical College, Huazhong University of Science and Technology, Wuhan, 430030 China; 2 Department of Clinical Laboratory, Institute of Translational Medicine, Renmin Hospital of Wuhan University, Wuhan, 430060 Hubei China; 3 Department of Neurobiology and Department of Neurology of the Second Affiliated Hospital, Zhejiang University School of Medicine, Hangzhou, China; 4 National Health and Disease Human Brain Tissue Resource Center, Hangzhou, China; 5 Co-innovation Center of Neuroregeneration, Nantong University, Nantong, 226000 China

**Keywords:** Alzheimer’s disease, Tau, Hippocampus, Episodic memory loss, MARK4

## Abstract

**Background:**

Intraneuronal accumulation of hyperphosphorylated tau is a hallmark of Alzheimer’s disease (AD). Given the significant correlation between tau pathology and memory loss in AD patients, identifying vulnerable brain regions, particularly susceptible neuron types in these regions, will advance our understanding of AD onset and shed light on therapeutic strategies to manage its progression.

**Methods:**

Immunofluorescent staining was employed to identify the brain regions and neuron types vulnerable to tau pathology in AD. A combination of chemogenetics, electrophysiological recording, in vivo Ca^2+^ recording, and a modified temporal-order discrimination behavior test was utilized to investigate the toxicity of tau accumulation to susceptible neurons in the dorsal part of the ventral hippocampus. Proteomics, phosphoproteomics, and molecular targeting were used to explore the underlying mechanisms of neuron susceptibility to tau accumulation in AD. The beneficial effects of microtubule affinity regulating kinase 4 (MARK4) knockdown and administration of DEPhosphorylation TArgeting Chimera (DEPTAC) were evaluated in AD mice with tau pathology.

**Results:**

In postmortem brains of AD patients, we observed robust accumulation of hyperphosphorylated tau in the anterior hippocampal CA1 region, particularly in its Calbindin1^−^ (Calb1^−^) neurons, as opposed to the posterior hippocampal CA1 region and Calb1^+^ neurons. The susceptibility of Calb1^−^ neurons to phospho-tau accumulation was also observed in P301L mice, especially in the dorsal part of ventral (anterior in human) hippocampal CA1 (dvCA1). In P301L mice, dvCA1 displayed distinct protein and phosphorylated protein networks compared with dorsal CA1, accompanied by overactivation of MARK4. Overexpressing human tau in Calb1^−^ neurons in the dvCA1 (dvCA1^Calb1−^ neurons) specifically impairs the temporal-order discrimination of objects. Meanwhile, tau accumulation significantly inhibited the excitability and firing patterns of dvCA1^Calb1−^ neurons associated with temporal-order discrimination. Knocking down MARK4 or reducing hyperphosporylated tau via DEPTAC in P301L mice significantly ameliorated AD-like tau pathology in dvCA1^Calb1−^ neurons and improved temporal-order discrimination of objects.

**Conclusion:**

These findings highlight the crucial role of dvCA1^Calb1−^ neurons in the early stage of tau pathology and demonstrate the potential of targeting phosphorylated tau through MARK4 knockdown or DEPTAC administration to counter the vulnerability of dvCA1^Calb1−^ neurons and, consequently, ameliorate episodic memory deficits in AD.

**Supplementary Information:**

The online version contains supplementary material available at 10.1186/s40035-025-00473-w.

## Background

Alzheimer’s disease (AD) is the most prevalent age-dependent neurodegenerative disorder, characterized by amyloid plaques and neurofibrillary tau tangle pathology [1–4]. Notably, tau pathology is strongly associated with episodic memory loss during the AD process than amyloid plaques [5–10]. Accordingly, screening for early cognitive dysfunctions and developing appropriate strategies to retard the progression of tau pathology are urgently needed for AD treatment.

The hippocampus is highly vulnerable to tau pathology [10–13]. Tau load detected by tau positron emission tomography, is associated with memory disorders [14, 15], and is particularly pronounced in the anterior and posterior parts of the hippocampus [14, 16, 17]. Animal experimental data revealed that exogenous overexpression of human tau in the hippocampus leads to Alzheimer-like spatial memory loss [13, 18, 19]. Molecules/pathways such as KEAP1 [20], STAT1 [21], and Galectin-3 [22], have been identified to mediate tau toxicity. Given the heterogeneity of the hippocampus along the dorsal-to-ventral axis in rodents, which corresponds to the posterior-to-anterior axis in humans [23, 24], understanding the neural susceptibility to tau pathology within hippocampal subregions could provide insights into the onset of AD. Moreover, screening for behavioral phenotypes corresponding to susceptible neuron types could help optimize cognitive function tests for patients with AD.

Hyperphosphorylated tau is a major protein component of neurofibrillary tangles in degenerative neurons in AD [25, 26]. Overactivation of protein kinases are responsible for tau hyperphosphorylation [27, 28]. According to the motif specificity, kinases for tau phosphorylation are divided into two major groups, the proline-directed protein kinases (PDPKs) and the nonproline-directed protein kinases (NPDPKs). PDPKs include extracellular signal-regulated kinase1/2, cell division cycle 2 kinase, cyclin-dependent kinase 2 (CDK2) and CDK5, while NPDPKs include calcium- and calmodulin dependent protein kinase II (CaMKII), protein kinase A (PKA), protein kinase C (PKC), casein kinase I and casein kinase II, and p110MARK. In addition to their independent roles, PDPKs and NPDPKs function synergistically to phosphorylate tau [29, 30], leading to disruption of a diffusion barrier for tau sorting [31], thereby causing mislocation of tau [31, 32], accelerating its aggregation [33–38], impairing synapses [39–41], disrupting autophagy [42–44], and priming inflammation [45–47]. However, the unique protein networks that contribute to the vulnerability of hippocampal neurons in response to tau accumulation remain unknown.

Protein phosphatases (PP) can dephosphorylate tau at multiple AD-related sites [48–50]. Notably, protein phosphatase 2A (PP2A) accounts for approximately 71% of the total tau phosphatase activity in the human brain, and its activity is significantly decreased in AD brains [50–53]. Recently, we have designed and synthesized a novel DEPhosphorylation TArgeting Chimaera (termed as DEPTAC) [54] to selectively facilitate the link between tau and Bα isoform of PP2A. It has proven effective in reducing AD-associated hyperphosphorylated tau both in vitro and in vivo [54]. However, the efficiency of DEPTAC in improving vulnerable hippocampal neurons in the presence of phospho-tau accumulation needs to be studied.

In the present study, we aim to: (1) identify vulnerable hippocampal neurons affected by tau pathology in AD; (2) uncover the molecular mechanisms underlying this vulnerability; and (3) evaluate the effectiveness of DEPTAC in improving vulnerable hippocampal neurons in the presence of tau pathology. To comprehensively address these objectives, we employed chemogenetics, electrophysiological recording, in vivo calcium imaging, proteomics, phosphoproteomics, and behavioral assays.

## Materials and methods

### Human brain sections

Two postmortem human brain samples containing anterior and posterior hippocampus were obtained from the China Brain Bank (CBB), i. e., National Brain Bank for Health and Disease, Hangzhou, China. All materials had been collected from donors from whom written informed consent for brain autopsy and the use of the material and clinical information for research purposes had been obtained by the brain bank. All procedures were in accordance with the 1964 Declaration of Helsinki and its later amendments. The clinical diagnosis of probable AD and Braak stages was confirmed by systematic neuropathological analyses as described previously [55, 56]. Exclusion criteria for control subjects were primary neurological/psychiatric disorders. Information of Braak degree, age, sex, postmortem delay and brain weight, is provided in Table S1.

### Animals

Adult C57BL/6 mice (6–8 weeks) were obtained from Beijing Vital River Laboratory Animal Technology Co., Ltd. Calb1-IRES2-Cre-D (B6.Cg-Calb1tm2.1(cre)Hze/J, Jax No. 028532) knock-in mice were purchased from Jackson Laboratory (Bar Harbor, ME), and crossed with the tdTomato reporter Ai9 (Jax No. 007905) or Ai14 (Jax No. 007914) strain to generate Calb1-IRES2-Cre-D::Ai9 or Calb1-IRES2-Cre-D::Ai14 mice with fluorescent labeling of Calbindin1 (Calb1)^+^ neurons. The genetic background of P301L tau transgenic pR5 mice was C57BL/6 × DBA2F1 [57]. The line was crossed at least for 10 generations onto a C57BL/6 background, and heterozygosity was maintained by wild-type C57BL/6 mice. The P301L tau transgenic pR5 mice were crossed with Calb1-IRES2-Cre-D::Ai14 mice to generate P301L::Calb1-IRES2-Cre-D::Ai14 mice. 3 × Tg-AD (B6;129-Tg (APPSwe, tauP301L)1Lfa *Psen1*^*tm1Mpm*^/Mmjax, Jax No. 004807) and nontransgenic mice (nTg, Jax No. 101045) were gifted by Dr. Xifei Yang (Laboratory of Modern Toxicology of Shenzhen, Shenzhen Center for Disease Control and Prevention, Shenzhen, China). hTau mice (Jax No. 005491) were generous gifts from Prof. Zhi-Hao Wang (Department of Neurology, Renmin Hospital of Wuhan University, Wuhan, China). All mice were housed at 24–25 °C under a 12-h light/dark cycle with food and water ad libitum. All the experiments were approved by the Animal Care and Use Committee of Huazhong University of Science and Technology.

### Behavioral test

All behavioral tests were conducted with adult male mice. Mice were handled and habituated to the open field once a day for three consecutive days prior to the experiments. Mice were allowed to acclimate to the room environment for 1 h before the behavioral experiments. The movement speed and the total distance mice travelled were recorded and analyzed by a video-tracking and data analysis tool (ANY-maze, O’Hara & Co., Tokyo, Japan). The odor was eliminated with 75% alcohol at experimental intervals.

#### Novel object recognition (NOR)

NOR experiment has a sample phase and a test phase. In the sample phase, two identical square objects (Objects A) were placed in the center of a 40 × 40 × 40 cm^3^ field arena, and mice explored the objects for 10 min. After 24 h, one of the objects was randomly selected to be replaced by a cylindrical object (Object B). In the test phase, mice were allowed to explore freely for another 10 min. Exploratory behavior was defined as having the snout within 2 cm proximity to the object. Standing or sitting on the object was excluded [58]. Videos were analyzed and scored by experimenters blinded to objects and animal identity. The object placement and objects were counterbalanced to minimize object and spatial bias [59]. The discrimination score was calculated as follows: discrimination score = (*t*_B _− *t*_A_)/(*t*_B_ + *t*_A_), where *t*_B_ and *t*_A_ represent the time the mice spent exploring Objects B and A, respectively.

#### Temporal-order recognition of objects

Temporal-order recognition experiment consisted of two sample phases and a test phase. In sample phase 1, two identical objects A were placed in the field arena (as mentioned in NOR), and mice were allowed to explore the objects for 10 min. One hour later, the mice underwent a second sample phase (sample phase 2), where they explored another set of two identical objects B for 10 min. One hour later, the mice were exposed to an object A from sample phase 1 (temporally-distant object) and an object B from sample phase 2 (temporally-recent object) for 10 min in the test phase. The exploratory behavior was defined as having the snout within 2 cm proximity to the object. Standing or sitting on the object was excluded [58]. Videos were carefully analyzed and scored by experimenters who were blinded to the objects and animal identities. The discrimination score was calculated as follows: discrimination score = (*t*_A _− *t*_B_)/(*t*_A_ + *t*_B_), where *t*_A_ and *t*_B_ represent the time the mice spent exploring Objects A and B, respectively.

### Stereotactic surgery

The adeno-associated viruses were obtained from Brain Case (Wuhan, China) and OBiO Technology (Shanghai, China). Calb1-Cre mice (20–30 g) were anesthetized with 1% sodium pentobarbital via intraperitoneal injection, and fixed in a stereotaxic frame. The skull was exposed after iodophor sterilization. Viral preparations (detailed information in Table S2) in volumes 200–300 nL were injected bilaterally into the dorsal part of ventral (anterior in human) hippocampal CA1 (dvCA1) (anteroposterior [AP] − 3.16 mm, mediolateral [ML] ± 3.0 mm, dorsoventral [DV] − 2.3 mm) or the dorsal CA1 (dCA1) (AP − 2.06 mm, ML ± 1.5 mm and DV − 1.5 mm) at a slow rate (50 nL/min) using a syringe pump. Heating pad was used to maintain body temperature after stereotactic injection. Behavioral experiments were conducted in mice 3–4 weeks after the virus injection.

For in vivo Ca^2+^ imaging experiments, 1:1 (*v*/*v*) mixture of AAV-CaMKIIa-DO-hTau-mCherry and AAV-EF1a-DO-GCaMP6f (or AAV-EF1a-DO-eGFP) viral preparations (300 nL) was injected into the dvCA1. After 3 weeks, an optical fiber (diameter = 1.25 mm (Ferrule), NA = 0.37, Newdoon, China) was implanted into the dvCA1 (AP − 3.16 mm, ML ± 3.0 mm and DV − 2.1 mm). One week later, the mice were allowed to explore freely, and the time exploring the object was manually marked. The optical fiber was connected to the fiber photometry system (Thinker Tech Nanjing Biotech Limited Co., Ltd, China). During recording, a 488 nm laser (0.01–0.02 mW) was delivered. MATLAB was used to analyze the data, generate the heatmaps and average Ca^2+^ traces. Calcium signal values were calculated as follows: Δ*F*/*F* (%) = (*F* − *F*_0_)/(*F*_0_ − *F*_offset_) × 100% [60], where *F*_0_ is the baseline signal in the absence of calcium transients and *F*_offset_ is the background fluorescence.

For microinjection of DEPTAC, a guide cannula (RWD, Shenzhen, China) was chronically implanted in the lateral ventricle (AP − 0.22 mm, ML ± 1.0 mm and DV − 2.5 mm), and adhered to the skull with adhesive and dental cement.

After completing all behavioral tests, viral injection sites or fiber/cannula placement was confirmed. Two animals with incorrect injection placements were excluded from the analyses.

### Drugs

P301L mice were repeatedly administered with DEPTAC (5 mmol/L, 1 μL each time) or saline through cannulas once every 3 days for a month. Mice were anesthetized continuously with 1.5% isoflurane and restricted in a stereotaxic instrument during drug administration. Behavioral experiments were conducted following the final DEPTAC administration, after which the mice were sacrificed.

For in vitro patch-clamp electrophysiology, clozapine-N-oxide (CNO, Sigma, C0832, Boston, MA) was dissolved in dimethyl sulfoxide (DMSO) and diluted with normal saline to a final concentration of 5 μmol/L. To silence dvCA1^Calb1−^ neurons in vivo, mice were intraperitoneally injected with CNO (4 mg/kg) 30 min before behavioral testing.

### Patch-clamp electrophysiology

Mice were transcardially perfused with cold cutting solution consisting of (in mmol/L): 228 sucrose, 26 NaHCO_3_, 11 glucose, 7 MgSO_4_, 2.5 KCl, 1 NaH_2_PO_4_, and 0.5 CaCl_2_ (280–290 mOsm, pH 7.3). The brain was quickly removed. Hippocampal slices (300 µm) were prepared and transferred to a holding chamber containing artificial cerebrospinal fluid (ACSF) consisting of (in mmol/L): 119 NaCl, 26 NaHCO_3_, 11 glucose, 2.5 KCl, 1 NaH_2_PO_4_, 1.3 MgSO_4_, and 2.5 CaCl_2_ (280–290 mOsm, pH 7.3). Slices were rested for 30 min at 34 °C and then at room temperature for at least 30 min prior to electrophysiological recordings. All solutions were saturated with 95% O_2_ and 5% CO_2_. Whole-cell patch-clamp recordings were performed using a micro-manipulator (Sutter, MP-225, Novato, CA) and an Axon Multiclamp 700B amplifier (Molecular Devices, Sunnyvale, CA). Data were acquired using the pClamp 11.2 software (Digidata 1550B, Axon, Sunnyvale, CA) with a sampling rate of 10 kHz. The recording electrode (4–7 MΩ) was filled with an internal solution containing (in mmol/L) 140 K-Gluconate, 20 HEPES, 10 KCl, 10 EGTA, 3 MgCl_2_, 1 CaCl_2_, 4 MgATP, and 5 NaGTP (290–300 mOsm, pH 7.3). Membrane potentials were held at − 70 mV. Current steps were then injected to generate action potentials (APs). Neuronal membrane capacitance and resting membrane potentials were measured within 2 min of break-in. Clampfit10 software was used for data analysis.

### In vitro Ca^2+^ imaging in brain slices

AAV-DO-GCaMP6-eGFP was injected into the dvCA1 of adult Calb1-Cre mice (300 nL). Preparation of acute brain slices (300 μm) was similar as that for electrophysiology. A slice was transferred to the recording chamber, which was continuously perfused with oxygenated ACSF (3 mL/min) at 26 °C. dvCA1 neurons were identified by a 40 × water-immersion objective of the Olympus BX51W1 microscope (Tokyo, Japan). The brain slices were perfused with 15 μmol/L KCl. For real-time Ca^2+^ signal recording, lights (488 nm) were delivered from an LED illumination resource (DC4100, Thorlabs, Newton, NJ). Data were analyzed using the ImageJ 1.53 (National Institutes of Health, Bethesda, MA) software. Ca^2+^ signals were calculated as Δ*F*/*F* using the following formula: Δ*F*/*F* = [(*F*_1_ − *B*_1_) − (*F*_0_ − *B*_0_)]/(*F*_0_ − *B*_0_). To plot calcium changes, the mean baseline signal was set to 0% [61]. *F*_0_ represents the baseline fluorescence intensity of the sample at the beginning of the experiment; *F*_1_ represents the fluorescence intensity measured after KCl treatment; *B*_0_ represents the baseline background fluorescence intensity at the beginning of the experiment; and *B*_1_ represents the background fluorescence intensity after KCl treatment.

### Tandem mass tag proteomics and phosphoproteomics

The same animals were used for proteomics and phosphoproteomics. Mouse brain slices were cut with a Vibratome VT1000S (Leica, Wetzlar, Germany) in ice-cold ACSF. For the dCA1, brain slices from AP − 1.46 mm to AP − 2.46 mm were collected, and the hippocampal CA1 region above the midline was manually dissected. For the ventral hippocampal CA1 (vCA1), brain slices spanning from AP − 2.92 mm to AP − 3.80 mm were collected, and the hippocampal CA1 region above the midline was manually dissected. The dvCA1 and dCA1 tissues were immediately snap-frozen in liquid nitrogen and then finely ground into powder. The resulting tissue powder was transferred to a 5-mL centrifuge tube, followed by addition of lysis buffer (8 mol/L urea, 1% protease inhibitor cocktail, 1% phosphatase inhibitor) and ice-cold sonication. For the phosphoproteomic experiments, 1% phosphatase inhibitor was included in the lysis buffer to facilitate phosphorylation analysis. All subsequent procedures, including centrifugation, digestion, labeling, and quenching, were carried out in accordance with the manufacturer’s instructions. The resulting MS/MS data were analyzed using the MaxQuant search engine (v.1.6.15.0). To ensure reliable identification, a false discovery rate < 1% was required for all reported proteins. Bioinformatics analyses were performed using Microsoft Excel and R statistical computing software. Fisher’s exact test was used to assess the significance of gene ontology (GO) enrichment for differentially expressed proteins, with a *P* value < 0.05 indicating statistical significance. Regarding the phosphoproteomic analysis, hyperphosphorylated and hypophosphorylated proteins were defined as follows: the intensities of modified peptides (I) were standardized and converted into relative quantitative values (R) for each sample. Pairwise comparisons were then conducted between groups, and the fold change (FC) was calculated as the ratio of mean intensities for each protein in the two sample groups. Student’s *t*-test was performed to determine the statistical significance of differences between groups applied to the relative quantitative values of each protein, with a typical threshold of *P* < 0.05 indicating significance. Proteins with a *P* value < 0.05 and a FC > 1.3 were classified as hyperphosphorylated proteins, while proteins with a *P* value < 0.05 and a FC < 1.0/1.3 were classified as hypophosphorylated proteins. Tandem mass tag proteomics and phosphoproteomics were conducted with the support of PTM Biolabs Inc. (Hangzhou, China).

### Cell culture

Primary hippocampal neurons were isolated from embryonic day 13 (E13)–E15 Sprague–Dawley (SD) rat embryos and maintained in neurobasal medium (21103049, Gibco, Waltham, MA), supplemented with 1% GlutaMAX, 2% B27 (Gibco, 17504044) and 1% penicillin–streptomycin (15140122, Thermo Fisher Scientific, Waltham, MA). The cultured neurons were transfected with pLenti-hSyn-MAPT-eGFP (OBio, Shanghai, China) at 4 days in vitro, and proteins were collected 1 week later.

### Western blotting

Brain tissues or primary hippocampal neurons were lysed and homogenized with RIPA lysis buffer (Beyotime, P0013B, Shanghai, China). Proteins were separated by 10% SDS/PAGE and transferred to a nitrocellulose membrane. After blocking in 5% nonfat milk at room temperature for 30 min, the membranes were incubated with primary antibodies overnight at 4 °C followed by incubation with the secondary antibody at room temperature for 1 h. Detailed information of the antibodies is provided in Table S3. Protein bands were visualized with the ECL system (ChemiScope 6000, Shanghai, China) and band intensity analyzed by ImageJ software.

### Immunofluorescence staining

Mice were deeply anesthetized with 1% sodium pentobarbital and subsequently perfused with saline, followed by ice-cold 4% paraformaldehyde (PFA) to fix the brain. The brains were post-fixed in 4% PFA overnight at 4 °C and then transferred to 25% and 30% sucrose solutions over a period of 3 days. Coronal sections of 30 μm in thickness were obtained using a Leica CM1860 cryostat. The brain slices were washed in PBS with 0.01% Triton-X, followed by incubation with primary antibodies at 4 °C for 15–18 h. Subsequently, the brain slices were incubated with the secondary antibody at 37 °C for 1 h. Finally, the sections were stained with DAPI (Beyotime, C1002, Shanghai, China).

For immunostaining on paraffin-embedded human brain section, the sections (7 µm) were deparaffinized in xylene and hydrated through descending ethanol concentrations. Antigen-retrieval was performed in boiling sodium citrate buffer (10 mmol/L) for 20 min. Brain slices were blocked with Sudan Black B (70% ethanol configuration) for 15 min to block autofluorescence. Four regions of the brain slices were randomly selected for comparative analysis.

Images were captured using an Olympus SV120 or LSM780 microscope and analyzed using the ImageJ software. For colocalization analysis, neurons that were DAPI^+^ and Calb1^−^ were identified as Calb1^−^ neurons. The proportion of colocalization with AT8 signals was then calculated. Information regarding the antibodies used is provided in Table S3.

### Statistical analysis

Statistical analysis was performed using the GraphPad Prism 8 and MATLAB softwares. Data were analyzed using unpaired two-tailed *t*-test, or one-way, two-way, or repeated-measures ANOVA with Tukey’s or Bonferroni’s multiple comparisons test. Data are expressed as mean ± SEM, and statistical significance was defined as *P* < 0.05.

## Results

### dvCA1^Calb1−^ neurons are vulnerable to phospho-tau accumulation in both AD patients and mouse models

**Fig. 1:**
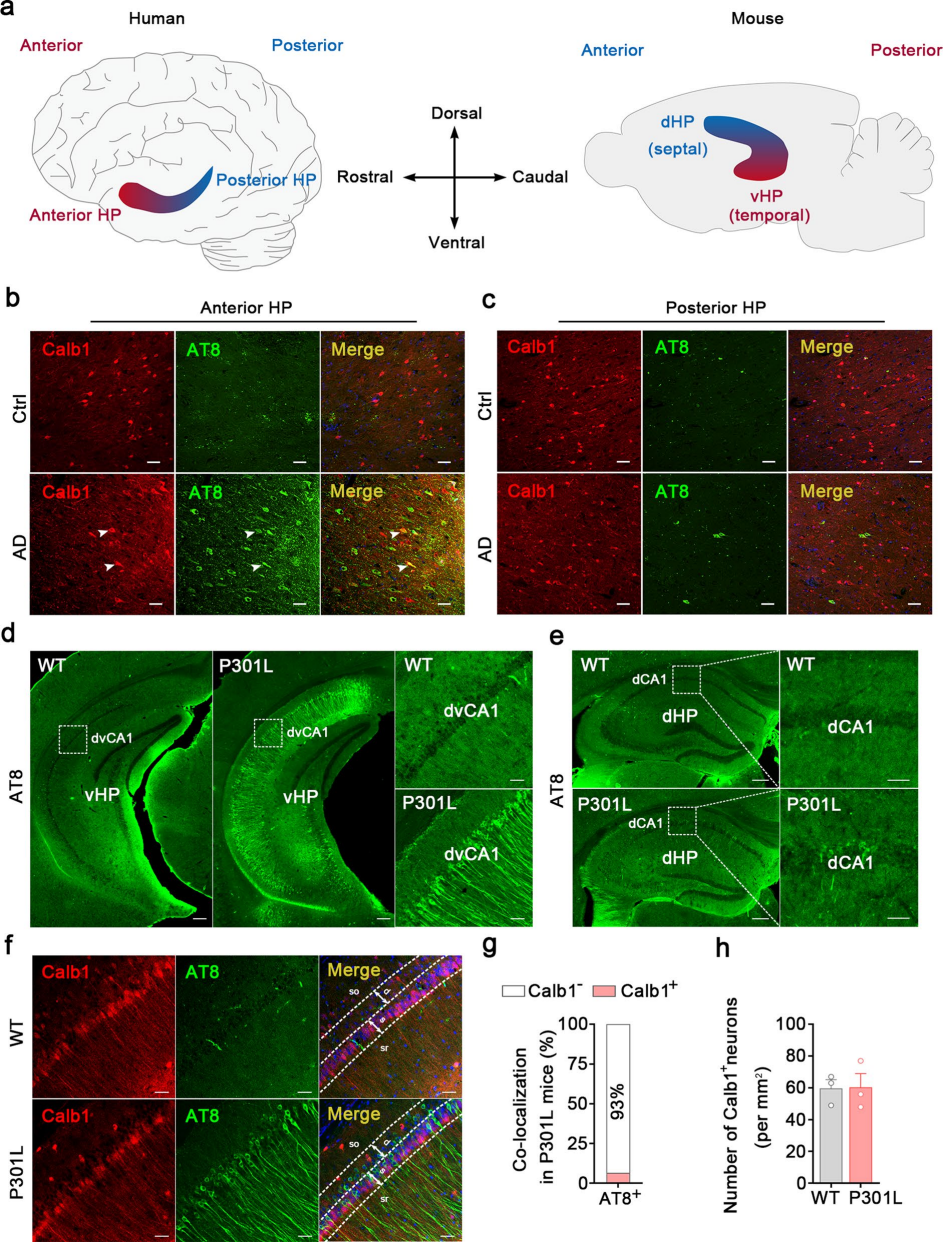
Hyperphosphorylated tau accumulates in hippocampal CA1^Calb1−^ neurons in AD. **a** Schematic of the longitudinal axes of the hippocampus in human and rodent brains. **b, c** Compared to non-demented controls, hyperphosphorylated tau (pTau, AT8) accumulated in the CA1 of anterior (aHP) and posterior (pHP) hippocampus of patients with AD. Hyperphosphorylated tau mainly accumulated in CA1^Calb1−^ neurons of aHP. Scale bars, 50 μm. Arrow heads indicated colocalization of Calb1 and pTau. **d, e** Representative images showing abundant hyperphosphorylated tau (pTau, AT8) in the dorsal part of ventral hippocampal CA1 (dvCA1, **d**), but not dorsal hippocampal CA1 (dCA1, **e**) of P301L mice. For whole hippocampus: scale bars, 500 μm (**d**) or 200 μm (**e**). For local magnification: scale bars, 100 μm (**d**) or 50 μm (**e**). **f** Representative images showing little colocalization of Calb1 with phosphorylated tau (AT8) in P301L mice. Scale bars, 50 μm. **g** Quantitative analysis showed that ~ 93% of AT8 were Calb1^−^ neurons in the dvCA1 of P301L mice. **h** There was no significant difference in the number of Calb1^+^ neurons between the two groups. Two-tailed unpaired *t*-test, *t* = 0.04517, *df* = 4, *P* = 0.9661. *n* = 3 mice per group. Data are presented as mean ± SEM

To investigate the differential susceptibility of hippocampal subregions to AD tau pathology, we used AT8 antibody to detect phosphorylated paired-helical-filament tau in the postmortem brains of AD patients, focusing on the anterior–posterior axis (which corresponds to the ventral-dorsal axis in rodents) of the hippocampus (Fig. 1a). We observed a larger number of AT8-immunoreactive (ir) neurons in the anterior hippocampal CA1 region compared to the posterior region (Fig. 1b, c). To further verify the susceptibility of rodent ventral hippocampal CA1 (vCA1) to tau pathology, we performed AT8 immunostaining in P301L and 3 × Tg-AD mouse models. P301L mice express the longest human tau isoform harboring the FTDP-17 P301L mutation under control of the neuron-specific mThy1.2 promoter [57]. At 3 months of age, an abundance of AT8-ir neurons was detected in P301L mice but not in wild-type (WT) mice (Fig. 1d). Along the dorsal-to-ventral axis of the hippocampus, AT8^+^ signals were found to accumulate predominantly in the vCA1 of P301L mice (Fig. 1d, e). This phenomenon was still evident when we compared dCA1 to dvCA1 (Fig. 1d, e). There were no significant differences in the number or morphology (i.e., branch points and branch length) of Iba1^+^ cells in the dvCA1 between 3-month-old P301L mice and WT mice (Fig. S1), suggesting no significant microglial activation in the dvCA1 despite accumulation of AD-like tau. Consistently, 3 × Tg-AD mice at 6 months of age displayed accumulation of AT8 signals in the dvCA1 but not dCA1 [62] (Fig. S2a). There were no AT8 signals in either dvCA1 or dCA1 of the nTg mice (Fig. S2b). These data indicate that dvCA1 is susceptible to tau accumulation, regardless of whether Aβ pathology is present.

Next, we aimed to explore which type of neurons in the dvCA1 is vulnerable to phospho-tau accumulation. Calb1 is a widely used marker for superficial neurons in the hippocampus [63–65]. By performing double immunostaining, we observed an abundance of AT8^+^ signals that were localized within cell bodies and neuronal processes of Calb1^−^ neurons, rather than Calb1^+^ neurons, in both the postmortem brains of AD patients (Fig. 1b, c) and 3-month-old P301L mice (Fig. 1f, g). There were no significant differences in the total number of neurons (Fig. S3) or Calb1^+^ neurons (Fig. 1h) in the dvCA1 between P301L mice and WT mice. Additionally, Western blotting results showed no significant differences in Calb1 protein levels between the WT and P301L groups (Fig. S4), indicating that there is no transformation of Calb1 + neurons into Calb1^−^ neurons at the early stage of AD-like tau pathology. To further investigate whether the observed phenotype is specific to AD tau or primary tauopathy, we employed hTau mice, which express only human tau isoforms without mouse tau [66, 67]. At 12 months of age, hTau mice exhibited substantial AT8 ^+^ signals in the dvCA1, compared to the dCA1 (Fig. S5a, b). No AT8 signals were detected in Tau-knockout mice (Fig. S5a, b). Co-staining revealed that approximately 21% of AT8^+^ signals were Calb1^+^ in 12-month-old hTau mice (Fig. S5b, c), indicating significant tau mislocation and accumulation within dvCA1 Calb1^−^ neurons. These findings align with observations in P301L mice (Fig. 1d–g) and further highlight the high susceptibility of dvCA1, particularly Calb1^−^ neurons in this region, to AD-like phospho-tau pathology.

### Tau accumulation in dvCA1^Calb1−^ neurons disrupts the temporal-order discrimination of objects

**Fig. 2:**
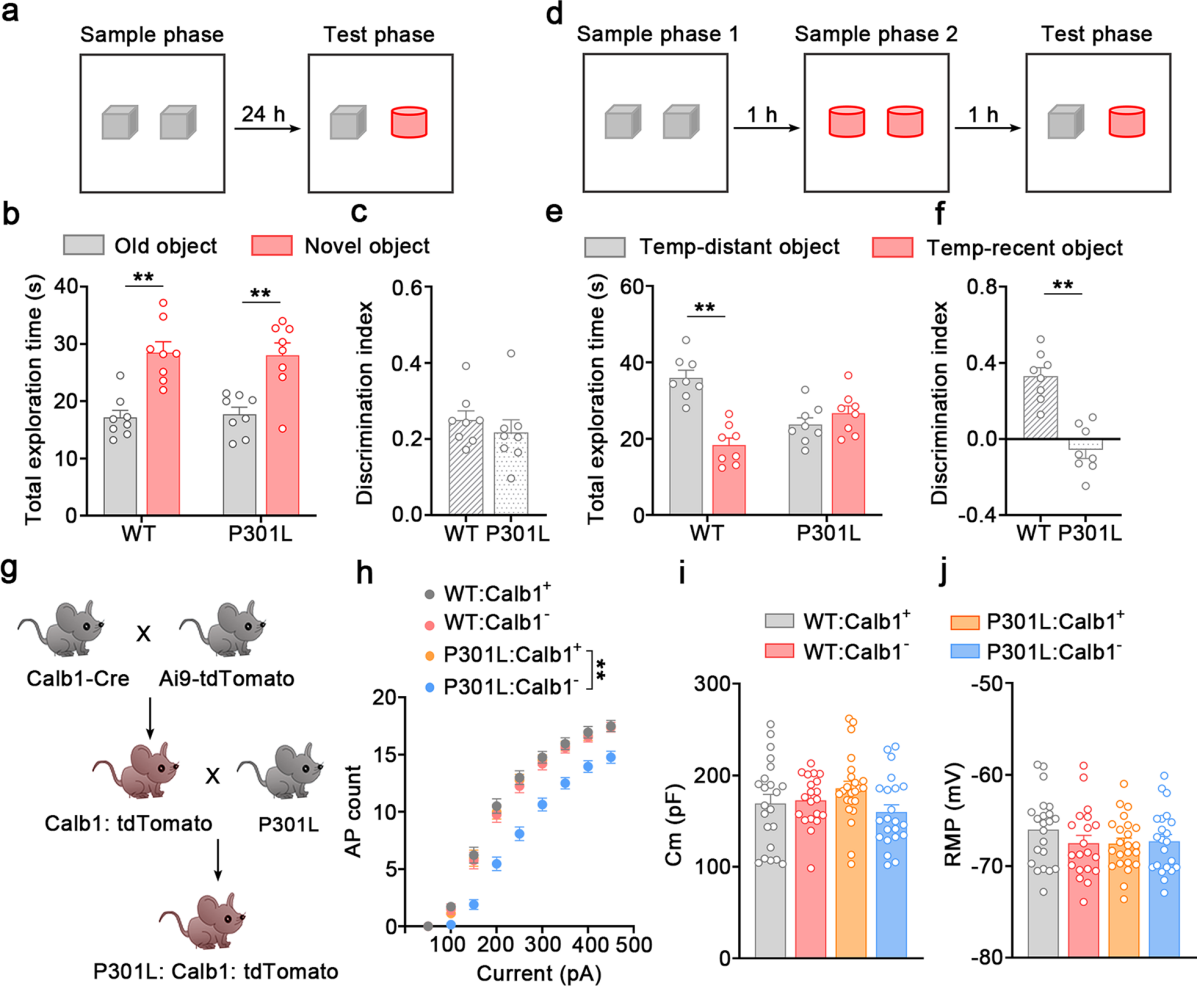
P301L mice exhibit temporal-order memory deficits and decreased excitability of dvCA1^Calb1−^ neurons. **a** Schematic of traditional novel object recognition test. **b** Both WT and P301L mice spent more time investigating novel object in the test. *F*_(1,28)_ = 0.3567, *P* = 0.5551, two-way ANOVA, Tukey’s multiple comparisons test. **c** Discrimination score did not differ significantly between the two groups.* t* = 1.128, *df* = 14, *P* = 0.2784, two-tailed unpaired *t*-test. *n* = 8 mice per group. **d** Schematic of temporal-order recognition. **e, f** P301L mice spent less time investigating the temporally distant object (**e**) and had a lower discrimination score (**f**) in the discrimination test. Two-way ANOVA, Tukey’s multiple comparisons test, *F*_(1,28)_ = 29.46, *P* < 0.0001 (**e**). Two-tailed unpaired *t*-test, *t* = 6.118*, df* = 14, *P* < 0.0001 (**f**).* n* = 8 mice per group. **g** Generation of P301L:Calb1:tdTomato mice. **h** The excitability of Calb1^−^ neurons in the dvCA1 of P301L mice was significantly decreased compared with WT group. For current–AP curves, fewer APs were induced by current injections in Calb1^−^ neurons of P301L mice. Interaction *F*_(24,664)_ = 7.586, *P* < 0.0001, Two-way repeated-measures ANOVA, Bonferroni’s multiple comparisons test. **i**, **j** No significant differences in the capacitance membrane (**i**) and resting membrane potential (**j**) were detected among the groups. One-way ANOVA, Tukey’s multiple comparisons test, interaction *F*_(3, 83)_ = 1.739, *P* = 0.1653 (**i**); interaction *F*_(3, 83)_ = 0.9269, *P* = 0.4315 (**j**). WT:Calb1^+^ mice: *n* = 22 neurons from 4 mice; WT:Calb1^−^ mice: *n* = 20 neurons from 4 mice; P301L:Calb1^+^ mice: *n* = 23 neurons from 4 mice; P301L:Calb1^−^ mice: *n* = 22 neurons from 4 mice. Data are presented as mean ± SEM., **P < 0.01. RMP, resting membrane potential

Considering the early onset of episodic memory decline in patients with AD [68–70], we performed a novel object recognition test in P301L mice (Fig. 2a). Both 3-month-old WT and P301L mice spent more time investigating the novel object than the familiar one (Fig. 2b). No statistical significance was detected between the two groups (Fig. 2b). The discrimination index of P301L group was comparable to that of WT mice (Fig. 2c), indicating intact cognitive ability of P301L mice to discriminate novel and familiar objects. Considering the contribution of temporal order in episodic memories, we modified the novel object recognition test and developed a novel paradigm, i.e., the temporal-order discrimination of objects (Fig. 2d). In another cohort of mice, no location preference was observed during two sample phases (Fig. S6). In the test phase, WT mice showed a significant preference for the temporally distant object in the discrimination test, evidenced by increased exploration time (Fig. 2e) and high discrimination index (Fig. 2f). However, this preference was not observed in the P301L mice (Fig. 2e). Moreover, the discrimination index of the P301L mice was much lower than that of WT mice (Fig. 2f).

Then, we performed whole-cell recording to evaluate the functional alterations of dvCA1^Calb1−^ neurons upon tau accumulation. Calb1-IRES2-Cre-D knock-in mice were crossed with the tdTomato reporter Ai9 mice to fluorescently label Calb1^+^ neurons, and the mice were further crossed with P301L mice to generate P301L: Calb1: tdTomato mice (Fig. 2g). As phospho-tau accumulated, the excitability of dvCA1^Calb1−^ neurons, but not dvCA1^Calb1+^ neurons, was dramatically decreased in the 3-month-old P301L mice (Fig. 2h). There was no significant difference in the membrane capacitance and membrane resting potential between the two neuronal types (Fig. 2i, j). Therefore, Calb1^−^ neurons in the dvCA1 were functionally inhibited upon tau accumulation.

**Fig. 3:**
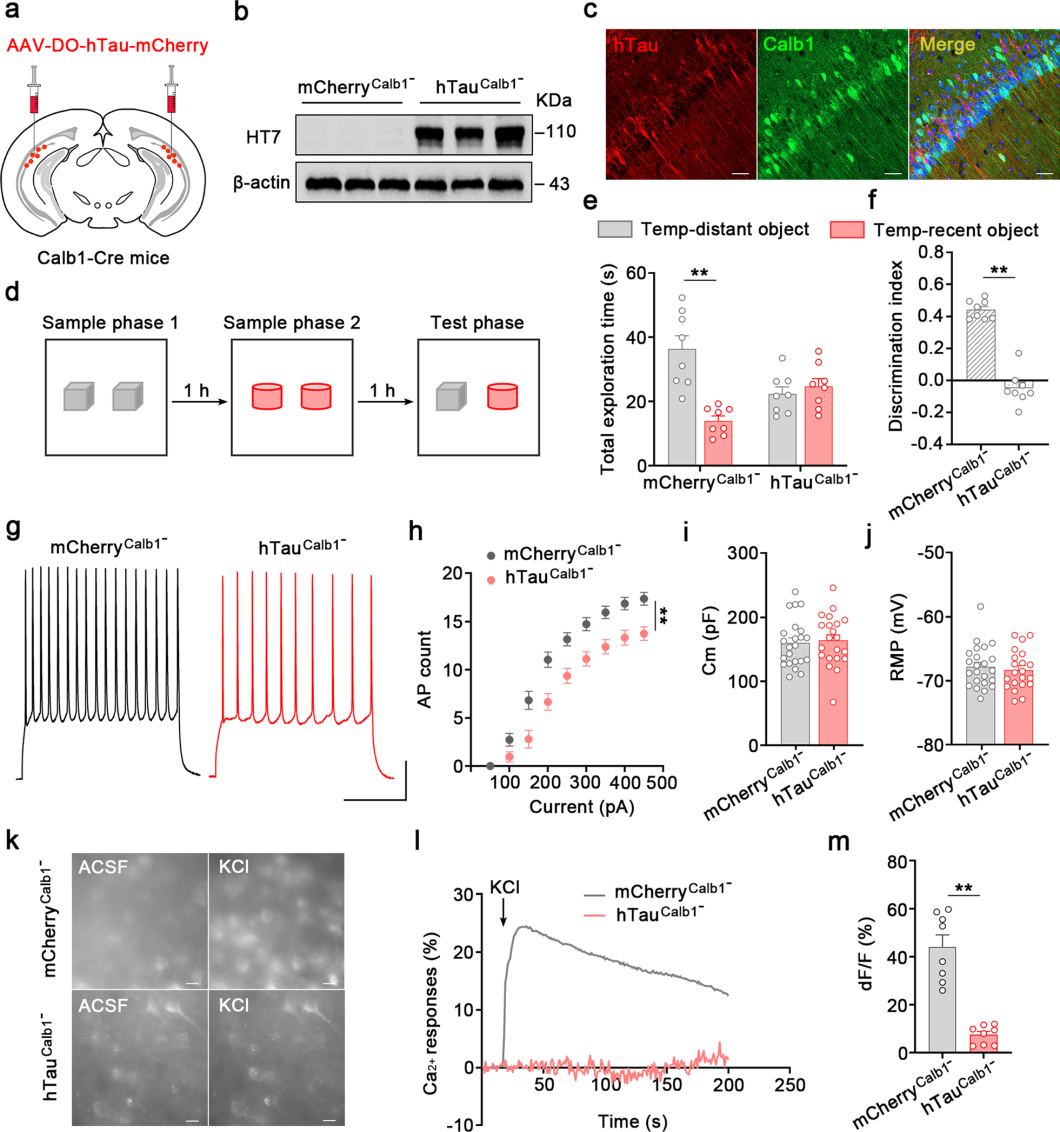
Tau accumulation in the dvCA1^Calb1−^ neurons disrupts the temporal-order discrimination of objects and decreases the excitability of dvCA1^Calb1−^ neurons. **a** Schematic of AAV-DO-hTau-mCherry viral injection into the dvCA1 of Calb1-Cre mice. **b** Expression of the exogenous hTau in the dvCA1^Calb1−^ neurons detected by Western blotting. **c** Representative images showing no colocalization of Calb1 and exogenously expressed hTau via Cre-off strategy. Scale bars, 50 μm. **d** Schematic of temporal-order recognition test. **e, f** Overexpression of hTau in the dvCA1^Calb1−^ neurons significantly disrupted object preference for the temporally distant object (**e**) and decreased the discrimination score (**f**) in the discrimination test. Two-way ANOVA, Tukey’s multiple comparisons test, *F*_(1,28)_ = 20.06, *P* = 0.0001 (**e**). Two-tailed unpaired *t*-test, *t* = 11.47*, df* = 14, *P* < 0.0001 (**f**).* n* = 8 mice per group. **g, h** The excitability of dvCA1-hTau^Calb1−^ neurons was significantly decreased. Representative traces of action potentials (APs) of Calb1^−^ neurons from mCherry^Calb1−^ and hTau^Calb1−^ groups following the injection of 250 pA currents (**g**). For current–AP curves, fewer APs were induced in hTau^Calb1−^ group than mCherry^Calb1−^ (**h**). Interaction *F*_(8,336)_ = 7.486, *P* < 0.0001, Two-way repeated-measures ANOVA, Bonferroni’s multiple comparisons test (**h**). **i**,** j** No significant differences of the capacitance membrane (**i**) and resting membrane potential (**j**) were detected between the two groups. Two-tailed unpaired *t*-test, *t* = 0.344, *df* = 42, *P* = 0.7326 (**i**); *t* = 0.5615, *df* = 42, *P* = 0.5774 (**j**). mCherry^Calb1−^: *n* = 23 neurons from 4 mice; hTau^Calb1−^: *n* = 21 neurons from 4 mice. **k–m** The intracellular Ca^2+^ signals in response to KCl (15 mmol/L) were significantly decreased in hTau^Calb1−^ group compared to mCherry^Calb1−^. GCaMP6 was introduced into Calb1^−^ neurons via Cre-off strategy. Scale bar, 20 μm. Quantification of the mean peak Ca^2+^ responses (**m**). Two-tailed unpaired *t*-test: *t* = 7.145, *df* = 14, *P* < 0.0001. *n* = 8 cells from 4 mice for each group. Data are presented as mean ± SEM., **P < 0.01. RMP, resting membrane potential

To investigate the causal effects of tau accumulation in the dvCA1^Calb1−^ neurons on the cognitive deficits, we overexpressed human tau (hTau, wild-type full-length human tau) in the Calb1^−^ neurons of dvCA1 via Cre-off strategy and conducted a series of behavior tests to evaluate cognitive ability. AAV-CaMKII-DO-mCherry or AAV-CaMKII-DO-hTau-mCherry was stereotaxically infused into the dvCA1 of Calb1-Cre mice at the age of 2 months, resulting in mCherry^Calb1−^ or hTau^Calb1−^ mice. Four weeks later, the expression of hTau, which was predominantly localized in the dvCA1^Calb1−^ neurons, was confirmed by immunofluorescence imaging and western blotting using the human-Tau-specific antibody HT7 (Fig. 3a–c). Many mCherry signals were observed within Calb1^−^ neurons in the dvCA1 (Fig. S7). In the novel object recognition test, no significant differences were observed between hTau^Calb1−^ and mCherry^Calb1−^ groups (Fig. S8). However, in the temporal-order recognition test, the dvCA1-hTau^Calb1−^ mice spent less time exploring the temporally distant object (Fig. 3d, e) and displayed a lower discrimination index (Fig. 3f) than the mCherry^Calb1−^ group. The temporal-order recognition deficits observed in the hTau^Calb1−^ mice were replicated when the activity of dvCA1^Calb1−^ neurons was inhibited using chemogenetics. AAV-CaMKII-DO-mCherry or AAV-CaMKII-DO-hM4Di-mCherry was stereotaxically infused into the dvCA1 of Calb1-Cre mice, resulting in mCherry or hM4Di expression in Calb1^−^ mice. Administration of CNO (5 μmol/L) significantly inhibited the firing of Calb1^−^ cells in the dvCA1, as confirmed by ex vivo electrophysiological recordings (Fig. S9a, b). Behaviorally, the hM4Di^Calb1−^ mice displayed identical exploration time for the temporally distant and temporally recent objects (Fig. S9c), and a lower discrimination index than the mCherry^Calb1−^ group (Fig. S9d). No significant difference was detected between hM4Di^Calb1−^ mice and mCherry^Calb1−^ mice in the traditional novel object recognition test (Fig. S9e, f).

To further address the specificity of dvCA1^Calb1−^ neuron involvement in tau-induced disruption of temporal-order discrimination of objects, we overexpressed hTau in the dCA1^Calb1−^ neurons (dCA1-hTau^Calb1−^ mice, Fig. S10a). The temporal-order discrimination ability was intact in the dCA1-hTau^Calb1−^ mice (Fig. S10b–d). Together, these data indicate that tau accumulation in the dvCA1^Calb1−^ neurons directly disrupts the temporal discrimination of objects.

### Tau accumulation impairs the firing patterns of dvCA1^Calb1−^ neurons associated with temporal-order discrimination

To investigate how accumulated tau affects the activity of dvCA1^Calb1−^ neurons, we performed in vitro patch-clamp recordings to evaluate changes in excitability following hTau overexpression. The intrinsic excitability of dvCA1^Calb1−^ neurons in response to step-current injection (Fig. 3g, h) was significantly decreased, with no changes in passive properties (Fig. 3i, j), indicating hypoactivity of dvCA1^Calb1−^ neurons in the presence of intracellular hTau accumulation. To further assess the excitability of dvCA1^Calb1−^ neurons in response to tau accumulation, we conducted intracellular Ca^2+^ recordings. GCaMP6f and hTau were introduced into dvCA1^Calb1−^ neurons via Cre-off strategy. The Ca^2+^ fluorescence signals of dvCA1^Calb1−^ neurons in brain slices were recorded before and after administration of KCl (15 mmol/L). In the mCherry^Calb1−^ group, Ca^2+^ signals were robustly increased in the presence of KCl (Fig. 3k–m). However, no significant fluctuations were recorded in the hTau^Calb1−^ group (Fig. 3k–m), indicating hypoactivity of dvCA1^Calb1−^ neurons in the presence of intracellular hTau accumulation.

**Fig. 4:**
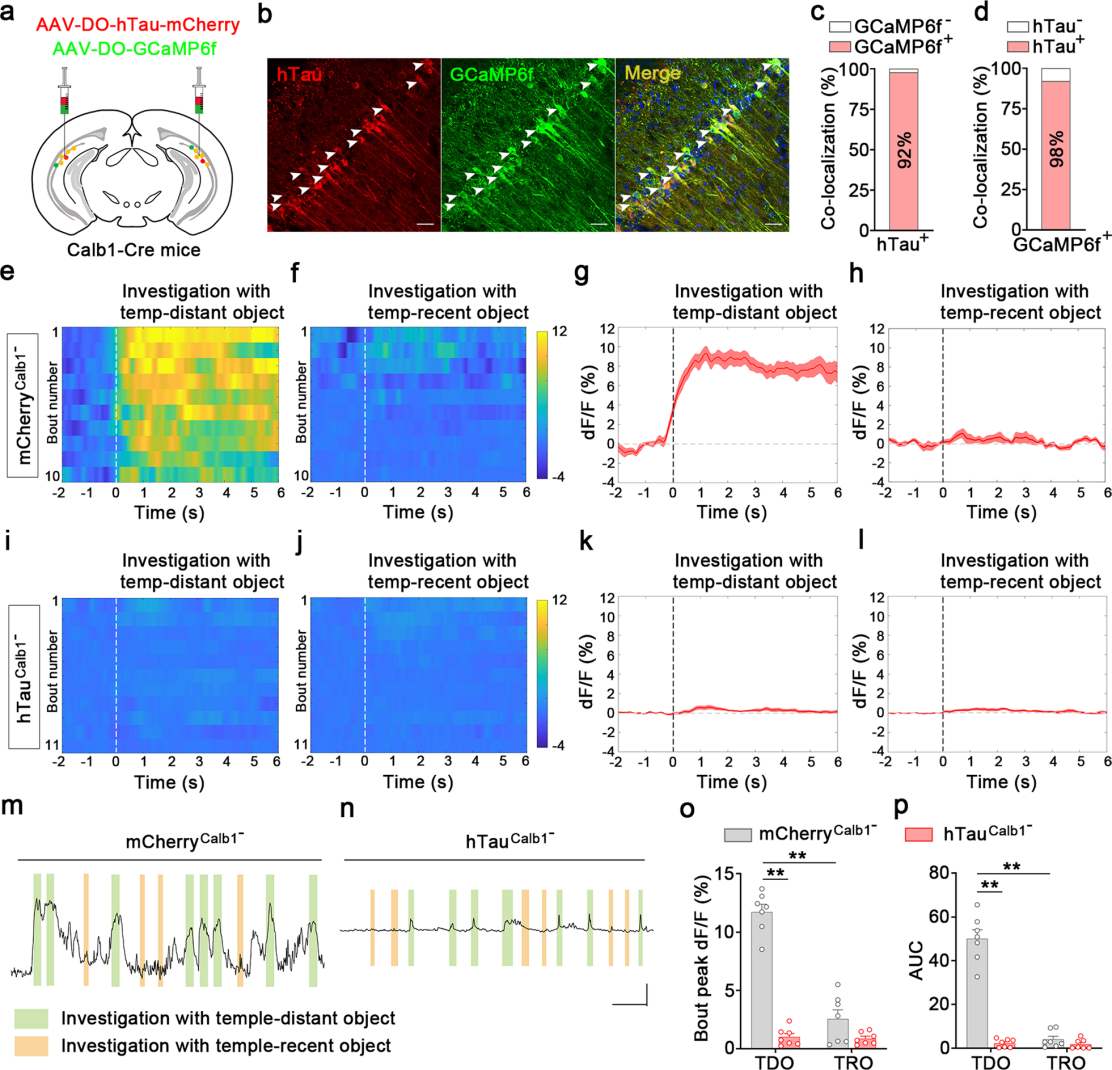
Tau accumulation disrupts firing patterns of dvCA1^Calb1−^ neurons. Tau accumulation specifically decreases GCaMP6f signals of dvCA1^Calb1−^ neurons during exploration of the temporally distant object. **a** Schematic of AAV-DO-hTau-mCherry and AAV-DO-GCaMP6f mixed virus injection into the dvCA1 of Calb1-Cre mice. **b** Representative images showing the colocalization of hTau and GCaMP6f. Arrows indicate co-labeled cells. Scale bar, 50 μm. **c** Quantitative analysis showed that 92% of hTau was colocalized with GCaMP6f. **d** Quantitative analysis showed that 98% of GCaMP6f was colocalized with hTau. **e, f, i, j** Heat maps of dvCA1^Calb1−^-GCaMP6f signals aligned to the start of the exploration event. **g, h, k, l** Per-bout stacked plots of dvCA1^Calb1−^-GCaMP6f signals aligned to the start of bout of exploration event. **m**, **n** Representative traces of GCaMP6f signals. **o**, **p** Quantitative analysis showed that tau accumulation in dvCA1^Calb1−^ neurons significantly decreased the peak Δ*F*/*F* (**o**) and AUC (**p**) of Ca^2+^ signals when mice were exploring the temporally distant object in the discrimination test. Two-way ANOVA, Tukey’s multiple comparisons test, interaction *F*_(1,24)_ = 70.5, *P* < 0.0001 (**o**); interaction *F*_(1,24)_ = 105.1, *P* < 0.0001,* n* = 7 mice per group. TDO, Temporally distant object; TRO, Temporally recent object. Data are presented as mean ± SEM. **P < 0.01 versus mCherry^Calb1−^ (TDO)

Then, we analyzed the real-time activity of dvCA1^Calb1−^ neurons during the temporal discrimination test. Again, GCaMP6f and hTau were co-expressed in the dvCA1^Calb1−^ neurons (Fig. 4a, b) using a Cre-off strategy, resulting in 92% hTau colocalized with GCaMP6f^+^ neurons and 98% GCaMP6f colocalized with hTau signals (Fig. 4c, d). The Ca^2+^ fluorescence signals were continuously recorded during the temporal-order discrimination test. A robust increase in Ca^2+^ signals was detected when mCherry^Calb1−^ mice were exploring a temporally distant object (Fig. 4e, g), but not a temporally recent object (Fig. 4f, h). These temporal discrimination-associated firings were dramatically inhibited in the hTau^Calb1−^ mice (Fig. 4i–n). Quantitative analysis revealed that the peak of Ca^2+^ signals and the area-under-the-curve (AUC) during interaction with a temporally distant object in the hTau^Calb1−^ group decreased to 8.2% and 3.3% of that in the mCherry^Calb1−^ group (Fig. 4o, p). Tau accumulation had no effect on eGFP signals in the dvCA1^Calb1−^ neurons (Fig. S11). These data demonstrate that tau accumulation suppresses dvCA1^Calb1−^ neurons during recognition of temporally distant object.

### MARK4 drives the vulnerability of dvCA1^Calb1−^ neurons to phospho-tau accumulation

**Fig. 5:**
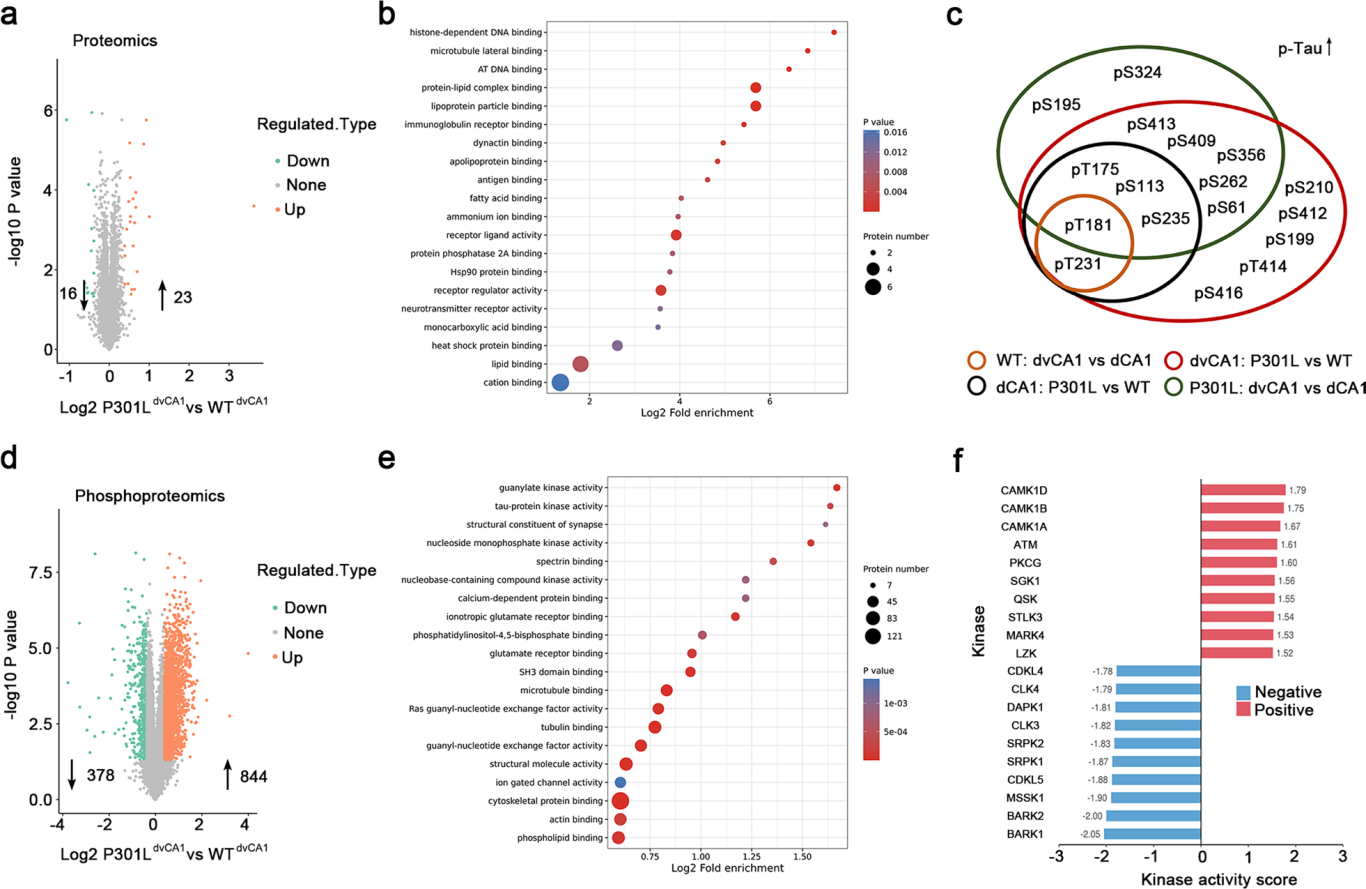
Proteomics and phosphoproteomics of dCA1 and dvCA1 in P301L and WT mice. **a, d** Volcano plots of proteomics (**a**) and phosphoproteomics (**d**) revealed protein changes in the dCA1 and dvCA1 between P301L and WT mice. **b, e** Fold enrichment analyses showed molecular functional enrichment clusters of differential proteins (**b**) and phosphorylated proteins (**e**) in the dCA1 and dvCA1 between P301L and WT mice. **c** Distinct phosphorylation sites of tau protein in the dCA1 and dvCA1 upon tau accumulation in P301L mice and WT mice. **f** Prediction of kinase activity in the dCA1 and dvCA1 of P301L versus WT mice

To explore the molecular mechanisms underlying the susceptibility of dvCA1^Calb1−^ neurons to tau accumulation, we dissected dvCA1 and dCA1 and performed proteomics and phosphoproteomics (Figs. 5 and S12 and S13). Proteomics identified 88 differential proteins out of 6628 analyzed, between dvCA1 and dCA1 in WT mice (Fig. S12a). Gene ontology (GO) enrichment analyses revealed that the top 3 enriched terms were related to metal ion binding, transporter activity and cation transmembrane transporter activity (Fig. S12b). In addition, 157 differential proteins were identified between dvCA1 and dCA1 in P301L mice, which were preferentially enriched in the molecular functions of regulation of calcium ion binding, structural molecule activity and structural constituent of ribosome (Fig. S12c). Phosphoproteomics identified 16,433 modified peptide sequences. Compared with dCA1, 937 and 490 modified proteins were hypophosphorylated and hyperphosphorylated, respectively, in the dvCA1 of WT mice (Fig. S13a). GO analysis revealed that the enriched terms were related with regulation of Ras guanyl-nucleotide exchange factor activity, Rho GTPase binding, tubulin binding, etc. (Fig. S13b). In the P301L mice, 1366 differentially modified proteins (844 hyperphosphorylated and 522 hypophosphorylated in the dvCA1 compared with dCA1) were related to actin binding, cation transmembrane transporter activity, tubulin binding, gated channel activity, microtubule binding, cation channel activity, ion gated channel activity, etc. (Fig. S13c). These data indicate the heterogeneity in terms of protein and phosphorylated protein networks along the anterior–posterior axis of hippocampal CA1 under both physiological and tau pathological conditions.

Proteomics revealed 39 differential proteins in the dvCA1 of P301L mice compared with WT mice. These proteins were enriched in cation binding, lipid binding, heat shock protein binding, etc. (Fig. 5a, b). In the dCA1, 63 differential proteins were identified between the WT and P301L groups (Fig. S12d). They were associated with heterocylic compound binding, organic cyclic compound binding, nucleic acid binding, RNA binding, etc. For phosphorylation protein networks, 844 and 378 proteins showed increased and decreased phosphorylation levels in the dvCA1 of P301L mice as compared with WT mice (Fig. S13a). These proteins were enriched in phospholipid binding, actin binding, cytoskeletal protein binding, etc. (Fig. 5e). However, in the dCA1, the differentially phosphorylated proteins between P301L and WT mice were related to cation channel activity, GTPase regulator activity, guanyl-nucleotide exchange factor activity, etc. (Fig. S13d).

Looking into the distinct phosphorylation sites of tau protein in the dvCA1 upon tau accumulation, we found that tau was hyperphosphorylated at pS61, pS113, pS199, pS210, pS235, pS262, pS356, pS409, pS412, pS413, pS416, pT175, pT181, pT231 and pT414 in P301L mice compared with WT mice (Fig. 5c). Taking into account the different phosphorylation sites of tau among comparisons of WT:dvCA1 versus WT:dCA1, P301L:dCA1 versus WT:dCA1, and P301L:dvCA1 versus P301L:dCA1, we speculate that pS413, pS409, pS356, pS262 and pS61 are closely related with phospho-tau accumulation in the dvCA1^Calb1−^ neurons.

**Fig. 6:**
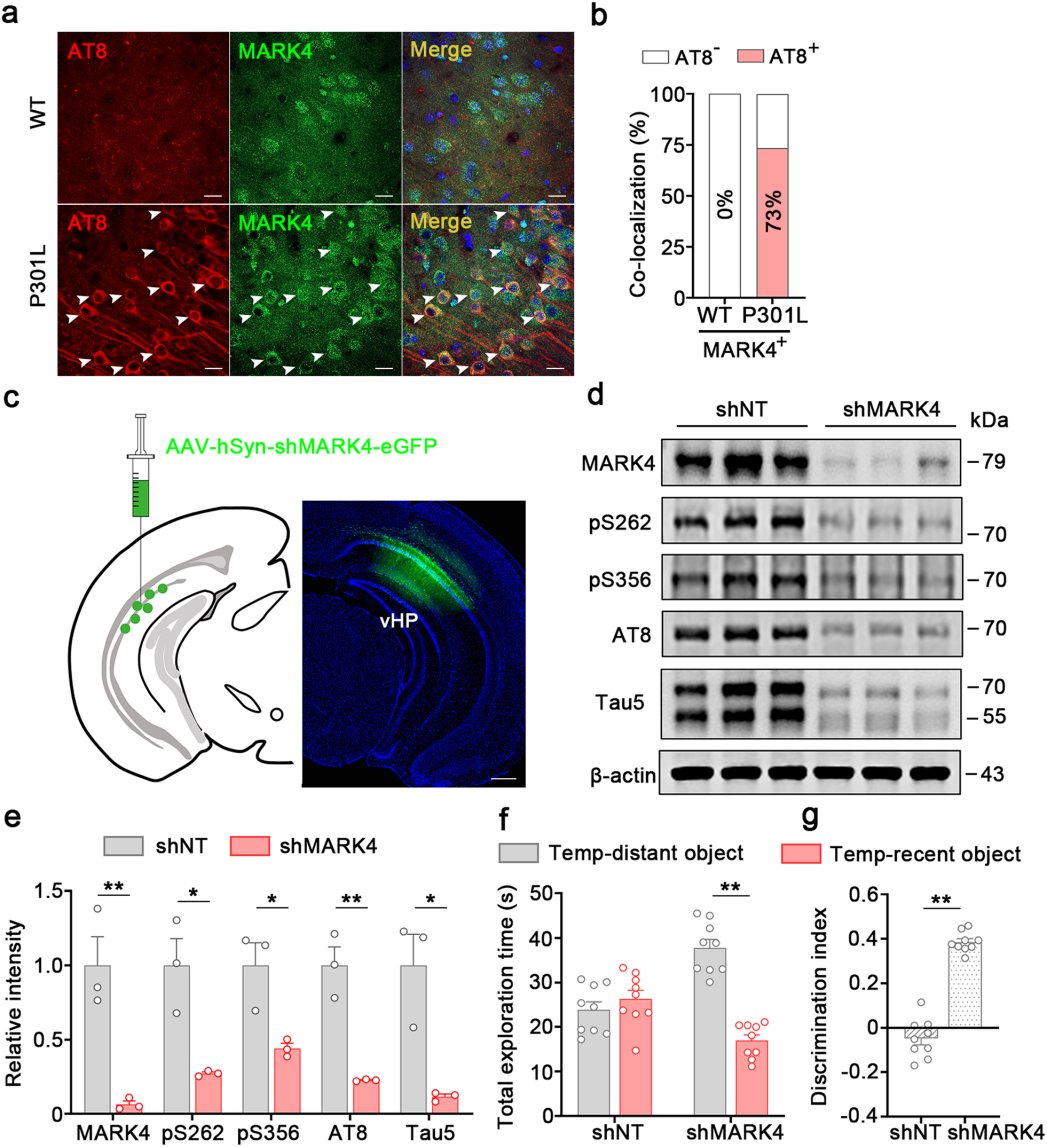
Knocking down MARK4 reduces the pathological tau load to improve temporal-order discrimination in P301L mice.** a** Representative images showing the colocalization of phosphorylated tau and MARK4. Arrows indicate co-labeled cells. Scale bars, 20 μm. **b** Quantitative analysis showed that 73% of MARK4^+^ neurons were colocalized with AT8 in P301L mice. **c** Representative images of AAV-hSyn-shMARK4-eGFP virus at the injection site. Scale bar, 500 μm. **d, e** Western blotting showed that knocking down MARK4 reduced phosphorylated tau (pS262, pS356 and AT8) and total tau (Tau5) in the dvCA1. Two-tailed unpaired *t*-test. MARK4:* t* = 4.817, *df* = 4, *P* = 0.0085; pS262:* t* = 4.016, *df* = 4, *P* = 0.0159; pS356:* t* = 3.57, *df* = 4, *P* = 0.0234; AT8:* t* = 6.223, *df* = 4, *P* = 0.0034; Tau5:* t* = 4.206, *df* = 4, *P* = 0.0136. *n* = 3 mice per group. **f, g** Knocking down MARK4 in the dvCA1 significantly increased exploration time with the temporally distant object (**f**) and improved the discrimination score (**g**) in the discrimination test. Two-way ANOVA, Tukey’s multiple comparisons test, *F*_(1,32)_ = 44.11*, P* < 0.0001 (**f**). Two-tailed unpaired *t*-test, *t* = 13.15*, df* = 16, *P* < 0.0001 (**g**). *n* = 9 mice per group. Data are presented as mean ± SEM., *P < 0.05, **P < 0.01

Overactivation of kinase(s) contributes to AD-like tau hyperphosphorylation [71–74]. Prediction analysis of kinase activity (Fig. 5f) revealed top 10 activated kinases for tau phosphorylation, including Calcium/calmodulin-dependent protein kinase type 1 (CAMK1), Serine-protein kinase ATM (ATM), Protein kinase C gamma type (PKCG), Serine/threonine-protein kinase Sgk1 (SGK1), Serine/threonine-protein kinase SIK3 (QSK), STE20/SPS1-related proline-alanine-rich protein kinase (STLK3), microtubule-affinity regulating kinase 4 (MARK4) and mitogen-activated protein kinase kinase kinase 13 (LZK) (Fig. 5f). Given that tau pS262 and pS356 are the preferential phosphorylation sites for MARK4 [75] and control axonal retention of tau in neurons [31], we speculate that MARK4 may be involved in phospho-tau accumulation in the dvCA1^Calb1−^ neurons. Then, we crossed Calb1-IRES2-Cre-D knock-in mice with the tdTomato reporter Ai14 mice to fluorescently label Calb1^+^ neurons. The Calb1-IRES2-Cre-D::Ai14 mice were further crossed with P301L mice to generate P301L::Calb1-IRES2-Cre-D::Ai14 mice. In control mice, few MARK4 signals were detected in Calb1^−^ neurons within the dvCA1 (Fig. S14a, b). In contrast, approximately 70% of Calb1^−^ neurons in the dvCA1 of P301L::Calb1-IRES2-Cre-D::Ai14 mice exhibited MARK4 ^+^ signals (Fig. S14a, b). By co-immunostaining, we found that ~ 73% of MARK4^+^ neurons were AT8^+^ in the dvCA1 of P301L mice (Fig. 6a, b). However, no significant colocalization of AT8^+^ and MARK4^+^ was observed in WT mice (Fig. 6a, b). These data strongly suggest an association between MARK4 and phospho-tau accumulation in dvCA1^Calb1−^ neurons.

Next, we aimed to explore whether knockdown of MARK4 could ameliorate phospho-tau accumulation in dvCA1^Calb1−^ neurons and improve temporal discrimination of objects. AAV-hSyn-shMARK4-eGFP or the control AAV-hSyn-shNT-eGFP (encoding a nontargeting shRNA) was infused into the dvCA1 of P301L mice (Fig. 6c). Reduction of MARK4 level was confirmed by Western blotting (Fig. 6d, e). Simultaneously, total tau (Tau5) and phosphorylated tau at pS262, pS356, or the AT8 site was significantly reduced in the dvCA1 of the P301L-shMARK4 group compared with the P301L-shNT group (Fig. 6d, e). Immunostaining revealed few AT8-ir signals in the dvCA1^Calb1−^ neurons after MARK4 knockdown (Fig. S15). Additionally, the protein levels of synapsin 1 (SYN1) and glutamate receptor 1 (GluR1) in the P301L group were significantly decreased compared to the WT group (Fig. S16a, b). No significant differences were observed for SYT1 (Synaptotagmin 1) and PSD95 (Postsynaptic density protein 95) between the groups (Fig. S16a, b). Following MARK4 knockdown, the protein levels of SYN1 and GluR1 were notably increased (Fig. S16c, d). These results suggest that MARK4 knockdown has beneficial effects in alleviating tau-induced synaptic dysfunction. Behaviorally, MARK4 knockdown dramatically increased the exploration time on the temporally distant object (Fig. 6f) and improved discrimination score during memory test (Fig. 6g), indicating an improvement of episodic memory by MARK4 knockdown. Together, our data reveal that MARK4 drives the vulnerability of dvCA1^Calb1−^ neurons to phospho-tau accumulation and impairment of temporal discrimination caused by phospho-tau accumulation.

### DEPTAC reverses phospho-tau accumulation in dvCA1^Calb1−^ neurons

To investigate whether dephosphorylation of tau proteins could ameliorate phospho-tau accumulation in the dvCA1^Calb1−^ neurons, we used the self-developed DEPTAC to selectively facilitate the binding of tau with PP2A-Bα [54]. A peptide with mutations in both the tau-binding and PP2A-Bα-recruiting domains (YQQYQAATAAAQGGSGS-KAVAVVRTPPASP-RRRRRRRR) was used as a control vehicle. In the primary hippocampal neurons instantly transfected with full-length human tau, 200 μmol/L DEPTAC was added. Twenty-four hours later, total tau and phosphorylated tau were measured by Western blotting (Fig. S17). Compared with the vehicle group, the levels of total tau (Tau5), human tau (HT7) and phosphorylated tau at pS199, pT205, pS262, pS396 and pS404 were significantly reduced in the DEPTAC group (Fig. S17).

**Fig. 7:**
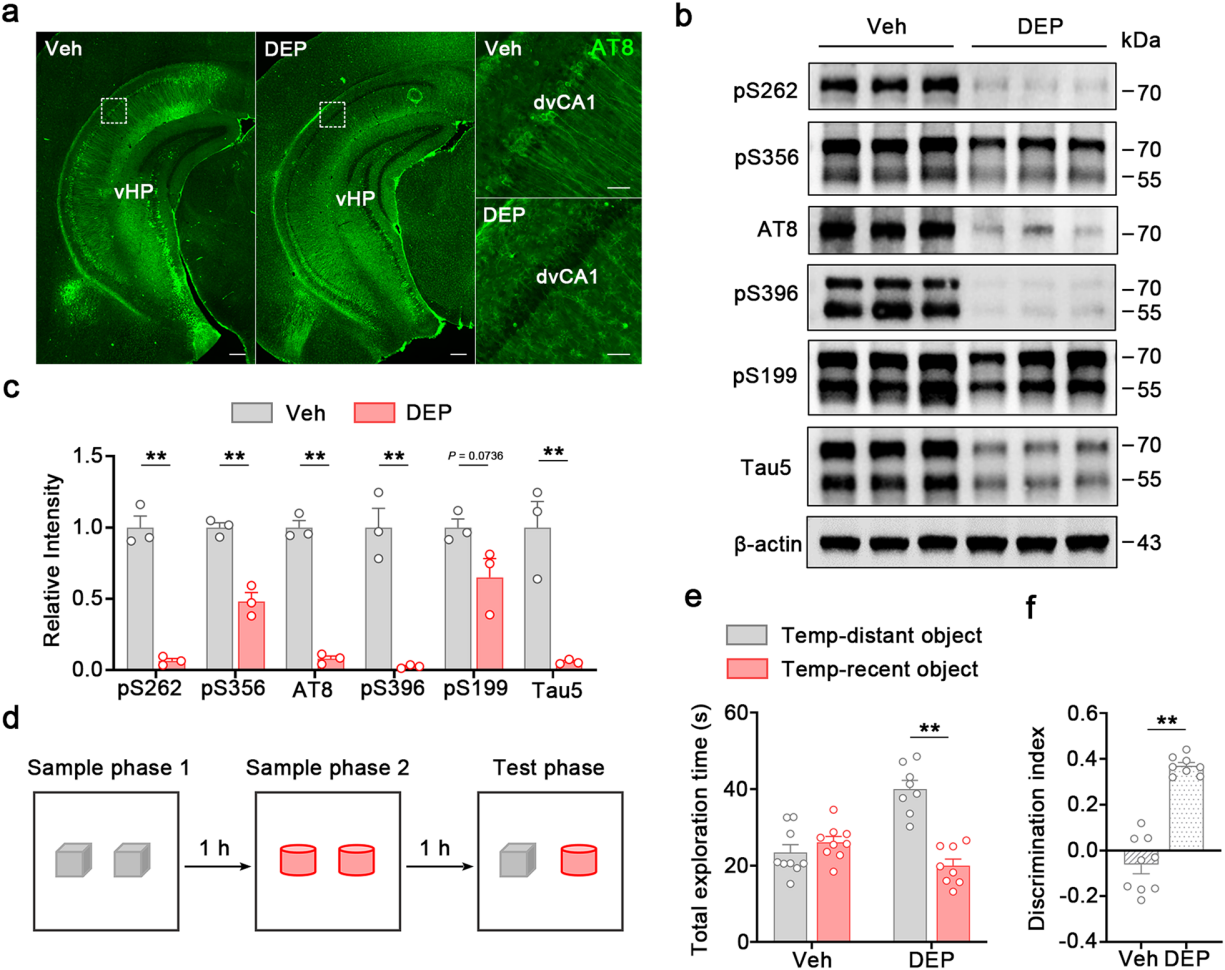
DEPTAC reduces pathological tau to improve temporal-order discrimination in P301L mice. **a** Representative images showing a robust reduction of phosphorylated Tau (pTau, AT8) in the dvCA1 of P301L mice after DEPTAC administration. Scale bars, 500 μm for whole hippocampus; 50 μm for local magnification. **b, c** DEPTAC treatment significantly reduced phosphorylated tau (pS262, pS356, AT8 and pS396) and total tau (Tau5) in the dvCA1 by Western blotting. Two-tailed unpaired *t*-test, pS262:* t* = 11.16, *df* = 4, *P* = 0.0004; pS356:* t* = 7.319, *df* = 4, *P* = 0.0019; AT8:* t* = 17.06, *df* = 4, *P* < 0.0001; pS396:* t* = 7.204, *df* = 4, *P* = 0.002; pS199:* t* = 2.409, *df* = 4, *P* = 0.0736; Tau5:* t* = 5.118, *df* = 4, *P* = 0.0069 (**b**).* n* = 3 mice per group. **d** Schematic of temporal-order discrimination of objects. **e, f** DEPTAC treatment effectively increased exploration time with the temporally distant object (**e**) and increased the discrimination score (**f**) in the discrimination test. Two-way ANOVA, Tukey’s multiple comparisons test, *F*_(1,30)_ = 35.48, *P* < 0.0001 (**e**). Two-tailed unpaired *t*-test, *t* = 9.605, *df* = 15, *P* < 0.0001 (**f**). Veh: *n* = 9 mice, DEP:* n* = 8 mice. Data are presented as mean ± SEM., *P < 0.05, **P < 0.01

Next, we tested whether DEPTAC can reduce phospho-tau accumulation in vivo. DEPTAC (5 mmol/L) was repeatedly delivered into the lateral ventricle of 3-month-old P301L mice for consecutive 30 days (once every 3 days, 1 μL each). Immunostaining revealed a significant reduction of phospho-tau (AT8-ir) accumulation in the dvCA1^Calb1−^ neurons (Fig. 7a). Western blotting further supported that the levels of total tau and tau phosphorylated at pS262, pS356, AT8 and pS396 were much lower in the DEPTAC group than in the vehicle group (Fig. 7b, c). Additionally, DEPTAC administration significantly increased the protein levels of SYN1 and GluR1 in the P301L mice compared to the controls (Fig. S18a, b), indicating that DEPTAC has beneficial effects in alleviating tau-induced synaptic dysfunction. Behaviorally, the P301L mice with DEPTAC administration spent more time investigating the temporally distant object and displayed a higher discrimination score than the vehicle-treated P301L mice (Fig. 7d–f). Together, these data highlight the effectiveness and advantages of DEPTAC in reducing phospho-tau accumulation in dvCA1^Calb1−^ neurons and in reversing memory impairment induced by tau accumulation.

## Discussion

In the present study, we focused on two issues: (1) the molecular mechanisms underlying the vulnerability of hippocampal subregions and neuronal types to tau pathology, and (2) promising strategies to improve these neurons in the presence of tau pathology. By immunostaining, we first uncovered that dvCA1^Calb1−^ neurons were susceptible to phospho-tau accumulation. As phospho-tau progressively accumulated, dvCA1^Calb1−^ neurons lost their firing patterns associated with temporal discrimination of objects. Meanwhile, the excitability of dvCA1^Calb1−^ neurons was significantly suppressed by tau accumulation. Proteomics and phosphoproteomics delineated distinct protein networks in hippocampal dvCA1 and dCA1 upon excessive tau load. Through prediction of kinase activity for tau and knocking down experiments, MARK4 upregulation was identified as a driving force for the susceptibility of dvCA1^Calb1−^ neurons to phospho-tau accumulation. Lastly, we found that the peptide DEPTAC efficiently improved dvCA1^Calb1−^ neurons in the presence of tau accumulation by reducing tau hyperphosphorylation and tau load. To the best of our knowledge, this is the first report to uncover the heterogeneity of hippocampal CA1 along the anterior–posterior axis. In addition to disclosing the vulnerability of dvCA1^Calb1−^ neurons to tau pathology, we identified MARK4 as the molecular target and showed evidence for the potential of peptide DEPTAC to reduce tau pathology in these neurons.

Hippocampus exhibits heterogeneity along the anterior–posterior axis [76, 77]. Functionally, the dorsal hippocampus is closely related to spatial memory [78, 79], while the ventral hippocampus is responsible for emotion [80, 81]. The hippocampal CA1 is the most vulnerable subregion to AD pathologies [82–84]. However, it remains unclear whether dorsal CA1 exhibits heterogeneity along the anterior–posterior and the superficial-deep axes. By proteomics and phosphoproteomics, we found differential protein networks between dCA1 and dvCA1 under physiological conditions. The differential proteins and phosphorylated proteins between dCA1 and dvCA1 in AD were enriched in GO terms of calcium ion binding and actin/microtubule-binding, respectively. Moreover, dvCA1^Calb1−^ neurons, but not dCA1^Calb1−^ neurons, displayed susceptibility to phospho-tau accumulation and contributed to tau-induced impairment of temporal discrimination of objects. It has been reported that the hippocampus, the prefrontal cortex [85, 86], the entorhinal cortex, and the perirhinal cortex, are critical for binding individual events or items to their temporal context in episodic memory [87]. Here, we revealed for the first time the heterogeneity of hippocampal CA1 under both physiological and pathological conditions, and identified a specific behavior phenotype associated with initial accumulation of phospho-tau in the hippocampal dvCA1^Calb1−^ neurons. Additionally, our immunostaining results from AD brain samples further support the high susceptibility of dvCA1^Calb1−^ neurons to tau pathology. To gain a more comprehensive understanding of neuronal susceptibility during the AD process, further investigations using brain samples from different Braak stages, ages, and genders are needed. Additionally, more experiments should be conducted using female AD mouse models. As the amygdala is also susceptible to tau pathology, as reported by Deters et al. in P301L mice [88], additional behavioral tests are warranted in future studies.

Accumulating evidence revealed that overactivation of kinases contributes to tau hyperphosphorylation. For example, phosphorylation of tau by GSK3β and CDK5 reduces tau affinity to microtubules [89]. Phosphorylation of tau at Ser214 by PKA also has a similar effect on tau–microtubule interactions [90]. Notably, phosphorylation of tau at Lys-Xaa-Gly-Ser (KXGS) motifs (particularly at Ser262 and Ser356) consistently suppresses interactions between tau and microtubules [91], and then breaks the retrograde diffusion barrier in the axon initial segment, leading to tau mislocation to the soma and dendrites [31]. MARK is a prominent kinase that phosphorylates tau at KXGS motifs [91]. Proteomics and phosphoproteomics in P301L mice detected hyperphosphorylated tau at Ser262 and Ser356 in the dvCA1, consistent with phospho-tau accumulation preferentially in the dvCA1^Calb1−^ neurons. Moreover, MARK4 was predicted to be overactivated and was observed to colocalize with phospho-tau in the dvCA1^Calb1−^ neurons of P301L mice as compared with WT mice. Knocking down MARK4 in the dvCA1 dramatically reduced phospho-tau accumulation and improved the temporal-order discrimination ability. In addition to the crucial roles of MARK4 and tau hyperphosphorylation at KXGS motifs in tau mislocation, we showed that MARK4 also plays a role in controlling the susceptibility of dvCA1^Calb1−^ neurons to tau pathology. Tau phosphorylation at Ser262 and Ser356 sites has been shown to promote further phosphorylation at the possible GSK3β phosphorylation epitopes, such as pS199, pS202 and pT205 [92]. This may be attributed to a structural change of tau induced by Ser262 phosphorylation, making tau more prone to further phosphorylation. Here, we detected more phosphorylated sites other than Ser262 and Ser356 in tau in P301L mice, and found significant dephosphorylation at those sites through MARK4 knockdown. Supporting previous findings, our data indicate that MARK4 acts an initial kinase to trigger the phosphorylation cascade of tau in the dvCA1^Calb1−^ neurons upon tau accumulation.

Boekhoorn et al. previously found increased long-term potentiation and improved cognitive performance in P301L mice at an age when tau phosphorylation is absent, despite tau overload in the hippocampus [93]. These data indicate that hyperphosphorylated tau, not tau, is the cause of the memory deficits. Therefore, targeting tau hyperphosphorylation presents a promising therapeutic strategy for AD [94]. Protein kinases have received significant attention as therapeutic targets in AD treatment research; however, most protein kinase inhibitors and PP2A activators have shown limited efficacy in numerous clinical trials [94]. The DEPTAC strategy offers significant advantages by recruiting PP2A to tau, thereby facilitating tau dephosphorylation while minimizing side effects associated with nonspecific dephosphorylation of unrelated proteins via PP2A [95]. In this study, both in vitro and in vivo experiments demonstrated the efficiency of DEPTAC. Moreover, DEPTAC preferentially targeted the dvCA1 as more phospho-tau was reduced in the dvCA1^Calb1−^ neurons than in other regions. Therefore, administration of DEPTAC may be promising for the treatment of AD via improving dvCA1^Calb1−^ neurons in the presence of phospho-tau accumulation.

## Conclusion

Our findings establish a causal relationship between phospho-tau accumulation in dvCA1^Calb1−^ neurons and impairment of temporal-order discrimination ability. Additionally, MARK4 plays a crucial role in the susceptibility of dvCA1^Calb1−^ neurons to phospho-tau accumulation. Knockdown of MARK4 and administration of DEPTAC to dephosphorylate tau offer promising approaches to slowing the progression of AD and related tauopathies.

## Supplementary Information

Additional file 1. **Figure S1.** No significant differences in the number and morphology of Iba1^+^ cells in the dvCA1 of 3-month-old P301L mice. **Figure S2.** Accumulation of hyperphosphorylated Tau in the dvCA1 of 6-month-old male 3×Tg AD mice. **Figure S3.** No neuron loss in the dvCA1 of 3-month-old male P301L mice. **Figure S4.** The Calb1 protein levels in the dvCA1 of 3-month-old male P301L mice are comparable to those in WT mice. **Figure S5.** Accumulation of hyperphosphorylated Tau in the dvCA1 of 12-month-old male hTau mice. **Figure S6.** No location preference during the sample phases of temporal order recognition. **Figure S7.** Efficiency of exogenously expressing hTau in Calb1- neurons via Cre-off strategy. **Figure S8.** Overexpression of hTau in dvCA1Calb1- neurons has no effect on novel object recognition. **Figure S9.** dvCA1Calb1^-^ neurons governs temporal order discrimination of objects. **Figure S10.** Overexpression of hTau in dCA1Calb1^-^ neurons has no effect on temporal order recognition. **Figure S11.** Tau accumulation in dvCA1Calb1^-^ neurons has no effects on eGFP signals during bouts of object exploration. **Figure S12.** Proteomics of dCA1 and dvCA1 in P301L and WT mice. **Figure S13.** Phosphoproteomics of dCA1 and dvCA1 in P301L and WT mice. **Figure S14.** MARK4 is significantly increased in Calb1^-^ neurons in the dvCA1 of P301L::Calb1-IRES2-Cre-D::Ai14 mice. **Figure S15.** Knocking down MARK4 reduces accumulation of hyperphosphorylated Tau in the dvCA1 of P301L mice. **Figure S16.** Knocking down MARK4 in the dvCA1 attenuates tau-induced synaptic protein loss in P301L mice. **Figure S17.** DEPTAC reduces pathological Tau in primary neurons instantly transfected with hTau. **Figure S18.** DEPTAC treatment increased synaptic proteins in P301L mice. **Table S1** The information of the human subjects. **Table S2** Virus strains and their applications. **Table S3** Antibody list.

Additional file 2. Full Western blots images.

## Data Availability

The datasets used and/or analyzed during the current study are available from the corresponding author on reasonable request.

## Abbreviations

ACSF: Artificial cerebrospinal fluid
AD: Alzheimer’s disease
Calb1: Calbindin1
Cm: Capacitance membrane
dCA1: Dorsal part of hippocampal CA1
DEPTAC: DEPhosphorylation targeting
dvCA1: Dorsal part of the ventral CA1
GCaMP6f: Genetically encoded calcium indicators
hTau: Human tau
MARK4: Microtubule-affinity regulating kinase 4
NOR: Novel object recognition
PP: Protein phosphatases
PP2A: Protein phosphatase 2A
pTau: Phosphorylated tau
