# Supplementary material for: Improving vulnerable Calbindin1− neurons in the ventral hippocampus rescues tau-induced impairment of episodic memory
Source: Transl Neurodegener. 2025 Mar 4;14:12. doi: 10.1186/s40035-025-00473-w (PMC11877784; doi:10.1186/s40035-025-00473-w)
Supplement: Supplementary file 1 — Additional file 1. Figure S1. No significant differences in the number and morphology of Iba1+ cells in the dvCA1 of 3-month-old P301L mice. Figure S2. Accumulation of hyperphosphorylated Tau in the dvCA1 of 6-month-old male 3×Tg AD mice. Figure S3. No neuron loss in the dvCA1 of 3-month-old male P301L mice. Figure S4. The Calb1 protein levels in the dvCA1 of 3-month-old male P301L mice are comparable to those in WT mice. Figure S5. Accumulation of hyperphosphorylated Tau in the dvCA1 of 12-month-old male hTau mice. Figure S6. No location preference during the sample phases of temporal order recognition. Figure S7. Efficiency of exogenously expressing hTau in Calb1- neurons via Cre-off strategy. Figure S8. Overexpression of hTau in dvCA1Calb1- neurons has no effect on novel object recognition. Figure S9. dvCA1Calb1- neurons governs temporal order discrimination of objects. Figure S10. Overexpression of hTau in dCA1Calb1- neurons has no effect on temporal order recognition. Figure S11. Tau accumulation in dvCA1Calb1- neurons has no effects on eGFP signals during bouts of object exploration. Figure S12. Proteomics of dCA1 and dvCA1 in P301L and WT mice. Figure S13. Phosphoproteomics of dCA1 and dvCA1 in P301L and WT mice. Figure S14. MARK4 is significantly increased in Calb1- neurons in the dvCA1 of P301L::Calb1-IRES2-Cre-D::Ai14 mice. Figure S15. Knocking down MARK4 reduces accumulation of hyperphosphorylated Tau in the dvCA1 of P301L mice. Figure S16. Knocking down MARK4 in the dvCA1 attenuates tau-induced synaptic protein loss in P301L mice. Figure S17. DEPTAC reduces pathological Tau in primary neurons instantly transfected with hTau. Figure S18. DEPTAC treatment increased synaptic proteins in P301L mice. Table S1 The information of the human subjects. Table S2 Virus strains and their applications. Table S3 Antibody list. [file 40035_2025_473_MOESM1_ESM.pdf]

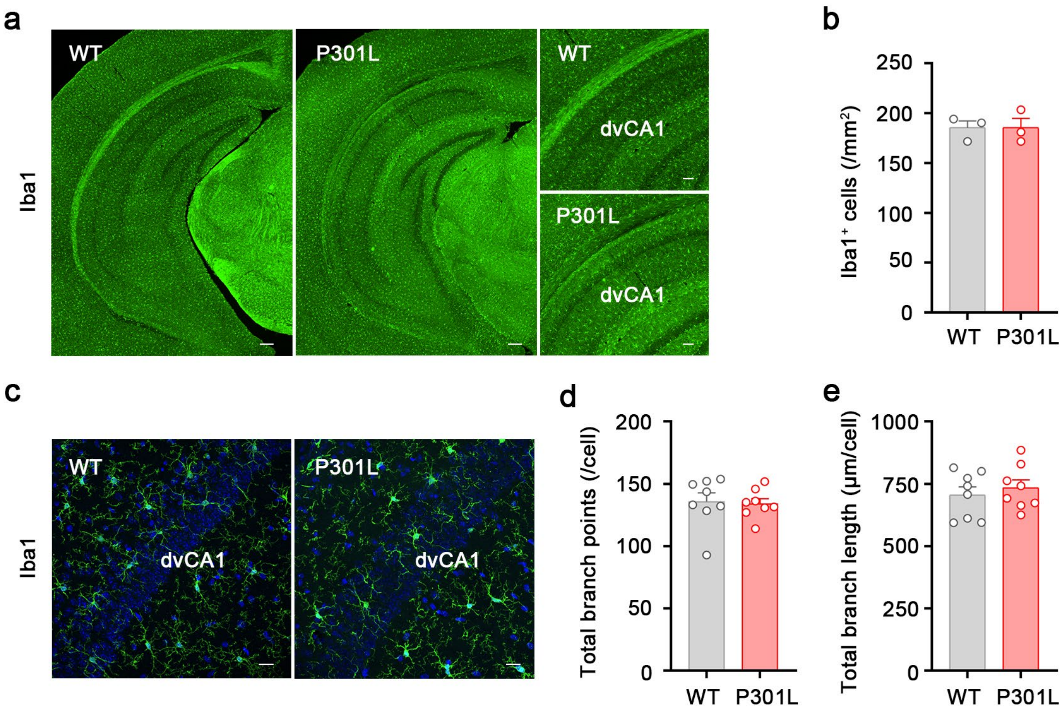

Figure S1. No significant differences in the number and morphology of Iba1$^+$ cells in the dvCA1 of 3-month-old P301L mice. (**a, b**) There was no significant difference in density of Iba1$^+$ cell between P301L mice and WT mice. Two-tailed unpaired t-test, $t = 0.000$, $df = 4$, $P > 0.9999$. $n = 3$ mice per group. For whole hippocampus: scale bar = 200 μm**.** For local magnification: scale bar =100 μm. (**c-e**) No significant difference was detected in total branch points (**d**) and total branch length (**e**) of Iba1$^+$ cells between P301L and WT mice. Scale bar = 20 μm. Two-tailed unpaired t-test, $t = 0.2043$, $df = 14$, $P = 0.8410$ (**d**); $t = 0.6486$, $df = 14$, $P = 0.5271$ (**e**). $n = 8$ sections from 3 mice per group. Data were presented as mean ± SEM.

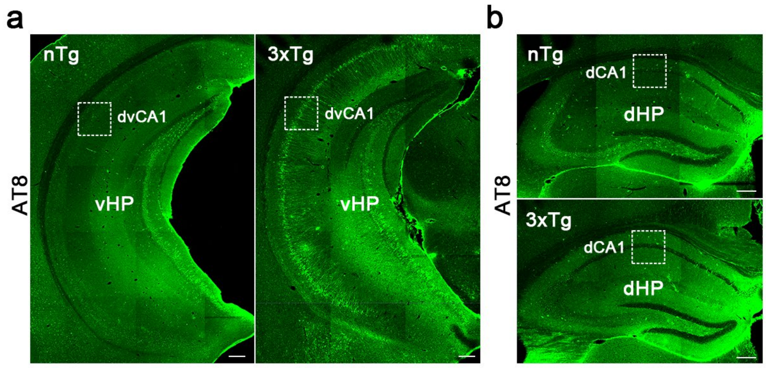

Figure S2. Accumulation of hyperphosphorylated Tau in the dvCA1 of 6-month-old male 3xTg AD mice. (**a**, **b**) Representative images showed abundant of hyperphosphorylated Tau (pTau, AT8) in the dorsal part of ventral hippocampal CA1 (dvCA1), not dorsal hippocampal CA1 (dCA1) of 3xTg AD mice. Scale bar = 500 μm (**a**) or 200 μm (**b**).

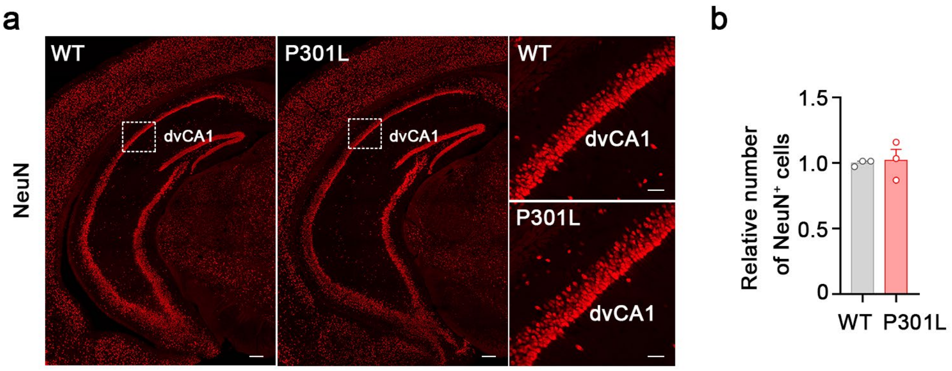

Figure S3. No neuron loss in the dvCA1 of 3-month-old male P301L mice. (a) Representative images of NeuN staining. For whole hippocampus: scale bar = 200 μm. For local magnification: scale bar = 50 μm. (**b**) No significant difference was detected in the number of NeuN-positive cells between 3-month-old P301L and WT mice. Two-tailed unpaired *t*-test, $t = 0.2433$, $df = 4$, $P = 0.8197$. $n = 3$ mice per group. Data are presented as mean ± SEM.

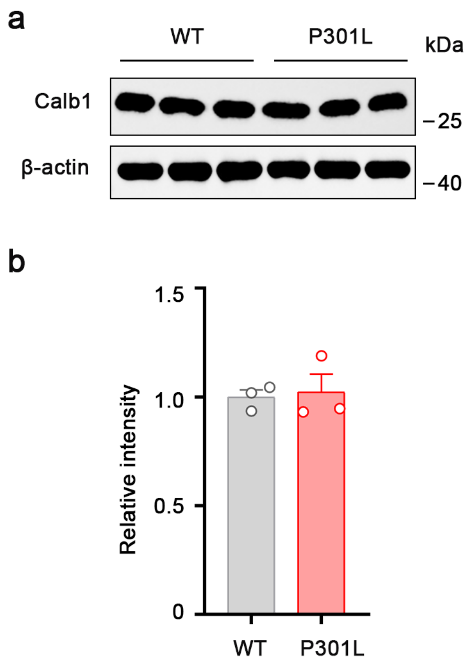

Figure S4. The Calb1 protein levels in the dvCA1 of 3-month-old male P301L mice are comparable to those in WT mice. (a, b) Western blot analysis showed no significant differences in Calb1 protein levels between the WT and P301L groups. Two-tailed unpaired *t*-test, $t = 0.2507$, $df = 4$, $P = 0.8144$. $n = 3$ mice per group. Data are presented as mean ± SEM.

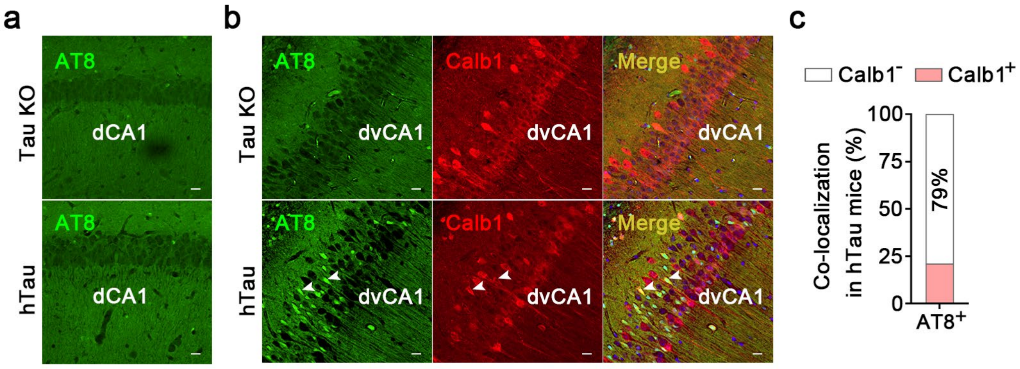

Figure S5. Accumulation of hyperphosphorylated Tau in the dvCA1 of 12-month-old male hTau mice. (**a, b**) Representative images showed abundant hyperphosphorylated Tau (pTau, AT8) in the dorsal part of ventral hippocampal CA1 (dvCA1, **b**), but not in the dorsal hippocampal CA1 (dCA1, **a**) of hTau mice. Scale bar = 20 μm. Arrow heads indicated colocalization. (**c**) Quantitative analysis revealed that approximately 21% of AT8-positive neurons in the dvCA1 of hTau mice were Calb1+.

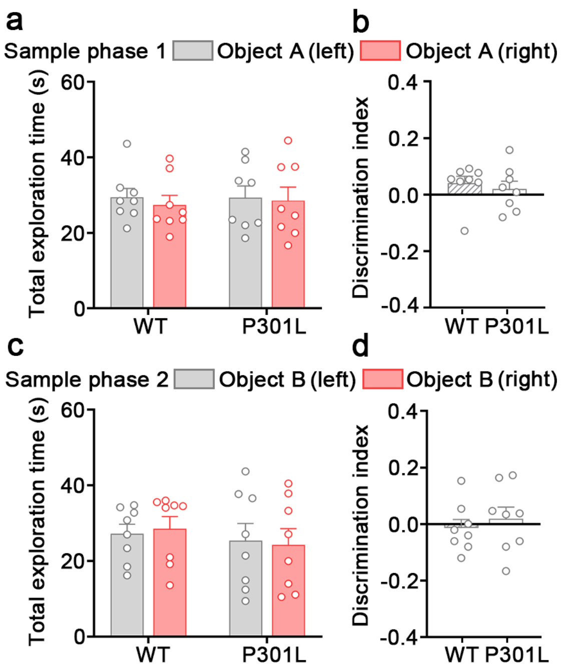

Figure S6. No location preference during the sample phases of temporal order recognition. (**a, c**) There was no difference in the exploration time of object A (**a**) or object B (**c**) in sample phase 1 and 2 between the two groups. Two-way ANOVA, Tukey's multiple comparisons test, interaction $F_{(1,\ 28)}$ = 0.04881, P = 0.8268 (**a**); interaction $F_{(1,\ 28)}$ = 0.3906, P = 0.5371 (**c**). (**b, d**) No difference was detected in discrimination score between the two groups. Two-tailed unpaired t-test, t = 0.5506, df = 14, P = 0.5906 (**b**); t = 0.6381, df = 14, P = 0.5337 (**d**). n = 8 mice per group. Data were presented as mean ± SEM.

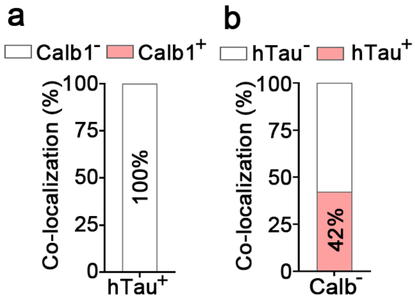

Figure S7. Efficiency of exogenously expressing hTau in Calb1$^-$ neurons via Cre-off strategy. (**a**) Quantitative analysis showed that 100% hTau$^+$ was colocalized with Calb1$^-$ neurons in the dvCA1. (**b**) Quantitative analysis showed that 42% Calb1$^-$ neurons were colocalized with hTau$^+$ in the dvCA1.

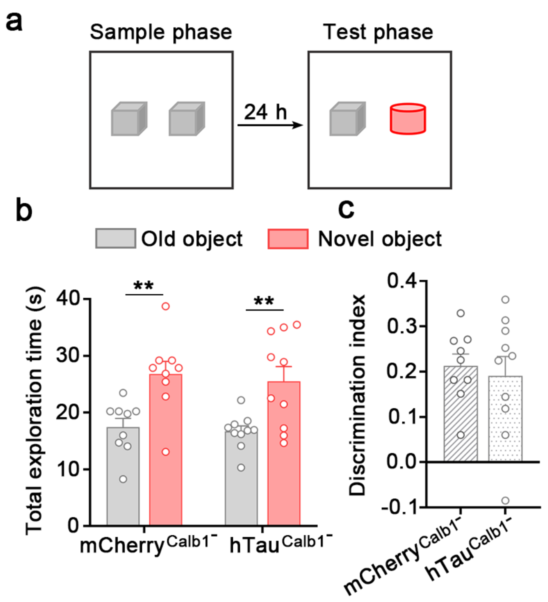

Figure S8. Overexpression of hTau in dvCA1^{Calb1-} neurons has no effect on novel object recognition. (**a**) Behavioral schematic of novel object recognition test. (**b**) Both mCherry^{Calb1-} and dvCA1-hTau^{Calb1-} mice spent more time investigating novel object in the test. Two-way ANOVA, Tukey's multiple comparisons test, $F_{(1, 34)}$ = 0.01571, P = 0.901. (**c**) No difference was detected in discrimination score between the two groups. Two-tailed unpaired t-test, t = 0.4188, df = 17, P = 0.6806. mCherry^{Calb1-}: n = 9 mice, hTau^{Calb1-}: n = 10 mice. Data were presented as mean ± SEM., *P < 0.05, **P < 0.01.

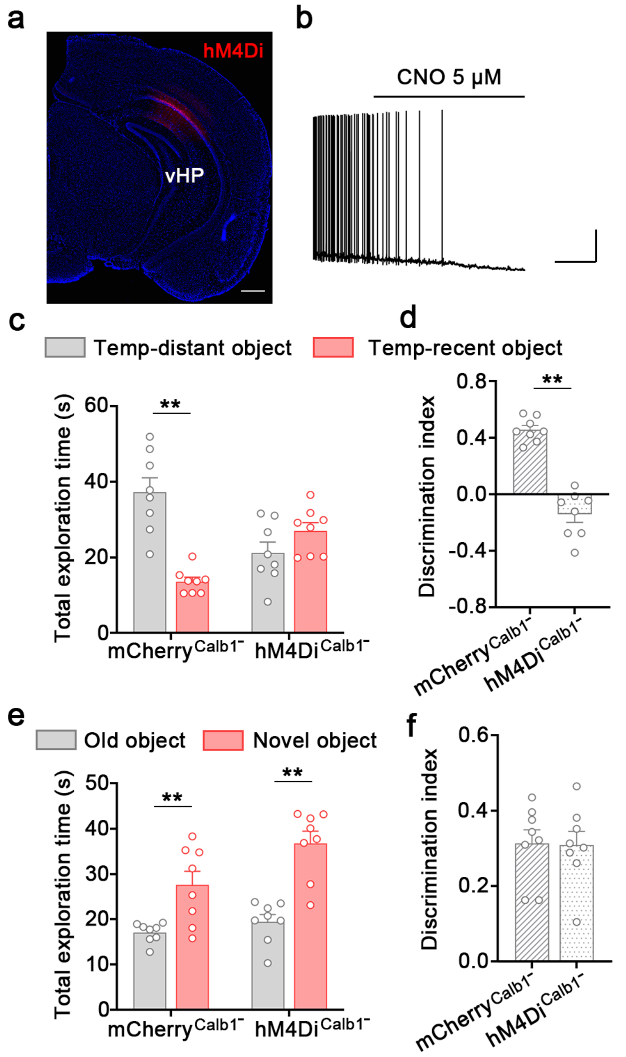

Figure S9. dvCA1^{Calb1-} neurons governs temporal order discrimination of objects. (**a**) Representative images of AAV-DO-hM4Di-mCherry virus into the dvCA1 of Calb1-Cre mice. Scale bar = 500 μm. (**b**) Representative trace recorded in current-clamp mode from a Calb- neuron in the dvCA1 that expressed hMD4i. Application of CNO abolished neuronal firing. Scale bars = 20 s, 20 mV. (**c, d**) Inhibition of dvCA1^{Calb1-} neurons significantly disrupted object preference for temp-distant object (**c**) and lowed the discrimination score (**d**) in the discrimination test. Two-way ANOVA, Tukey's multiple comparisons test, $F_{(1,28)} = 30.57$, $P < 0.0001$ (**c**). Two-tailed unpaired *t*-test, $t = 9.227$, $df = 14$, $P < 0.0001$ (**d**). (**e**) Both mCherry^{Calb1-} and hM4Di^{Calb1-} mice spent more time investigating novel object in the test. Two-way ANOVA, Tukey's multiple comparisons test, $F_{(1,28)} = 2.425$, $P = 0.1307$. (**f**) No difference was detected in discrimination score between the two groups.

Two-tailed unpaired *t*-test, $t = 0.09514$, $df = 14$, $P = 0.9256$. $n = 8$ mice per group. Data were presented as mean ± SEM., $^{**}P < 0.01$.

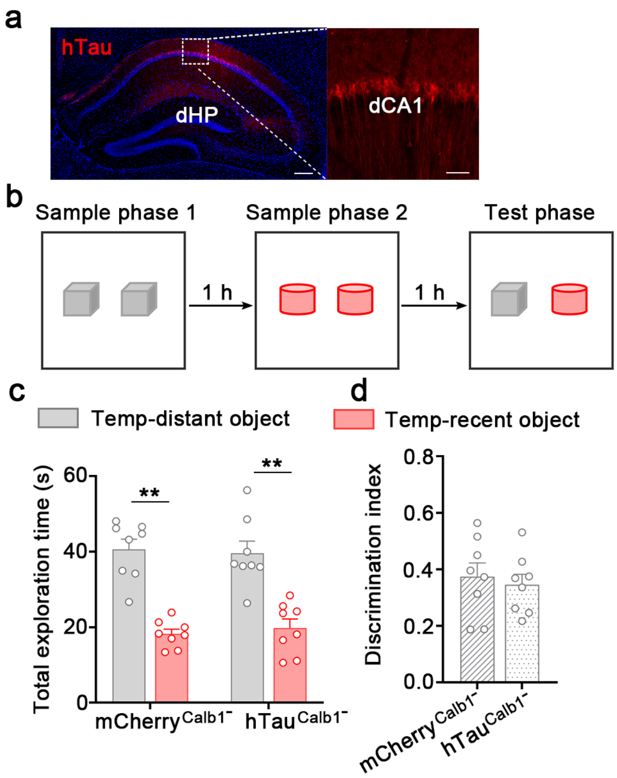

Figure S10. Overexpression of hTau in dCA1^{Calb1-} neurons has no effect on temporal order recognition. (**a**) Representative images of AAV-CaMKII -DO-hTau-mCherry virus into the dCA1 of Calb1-Cre mice. Left: dorsal hippocampus, scale bar = 200 µm; right: local magnification image of the dCA1, scale bar = 50 µm. (**b**) Behavioral schematic of temporal order recognition test. (**c**) Both dCA1-mCherry^{Calb1-} and dCA1-hTau^{Calb1-} mice spent more time investigating temp-distant object in the test. Two-way ANOVA, Tukey's multiple comparisons test, $F_{(1, 28)} = 0.2713$， $P = 0.6066$. (**d**) No difference was detected in discrimination score between the two groups. Two-tailed unpaired *t*-test, $t = 0.4553$, $df = 14$, $P = 0.6558$. $n = 8$ mice per group. Data were presented as mean ± SEM., $^{**}P < 0.01$.

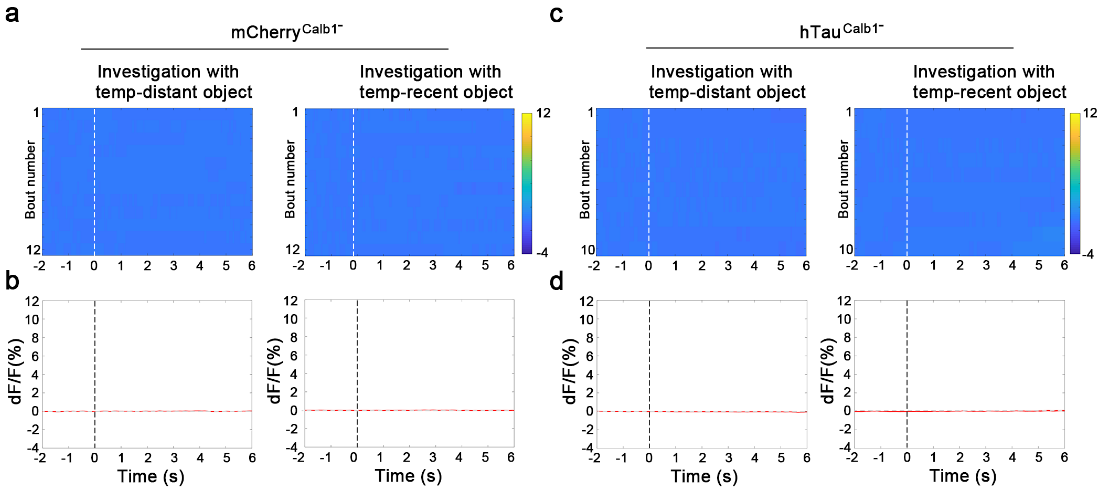

Figure S11. Tau accumulation in dvCA1^{Calb1-} neurons has no effects on eGFP signals during bouts of object exploration. Heat maps (**a, c**) and per-bout stacked plots (**b, d**) of eGFP signals were aligned to the start of each bout of exploration event in mCherry^{Calb1-} and hTau^{Calb1-} groups. *n* = 5 mice per group.

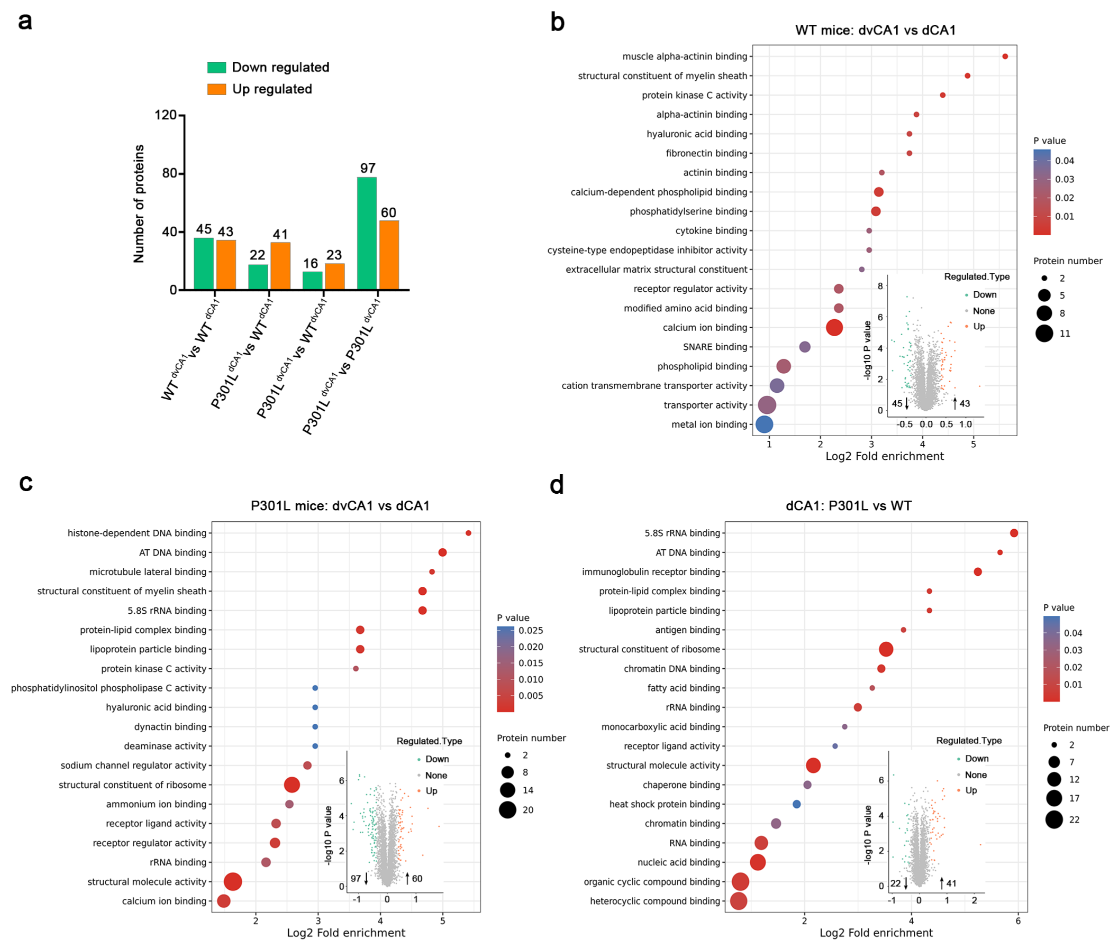

Figure S12. Proteomics of dCA1 and dvCA1 in P301L and WT mice. (a) Proteomics revealed the differential proteins in the dCA1 and dvCA1 between P301L and WT mice. (**b, c**) Fold enrichment analyses showed molecular functional enrichment cluster and volcano plot of differential proteins between the dCA1 and dvCA1 of WT (**b**) and P301L (**c**) mice. (**d**) Fold enrichment analyses showed molecular functional enrichment cluster and volcano plot of differential proteins in the dCA1 between WT and P301L mice.

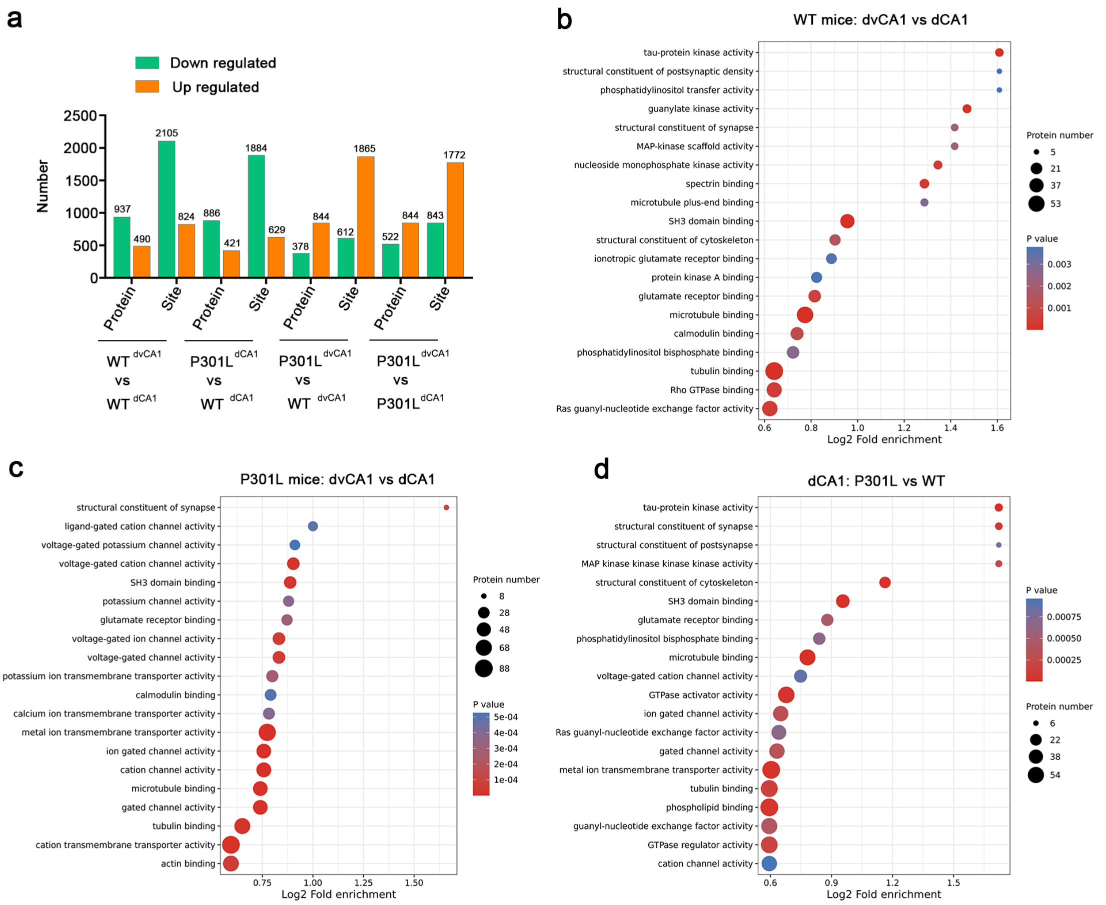

Figure S13. Phosphoproteomics of dCA1 and dvCA1 in P301L and WT mice. (a) Phosphoproteomics revealed the differential phosphorylated proteins in the dCA1 and dvCA1 between P301L and WT mice. (**b, c**) Fold enrichment analyses showed molecular functional enrichment cluster of differential phosphorylated proteins between the dCA1 and dvCA1 of WT (**b**) and P301L (**c**) mice. (**d**) Fold enrichment analyses showed molecular functional enrichment cluster of differential phosphorylated proteins in the dCA1 between WT and P301L mice.

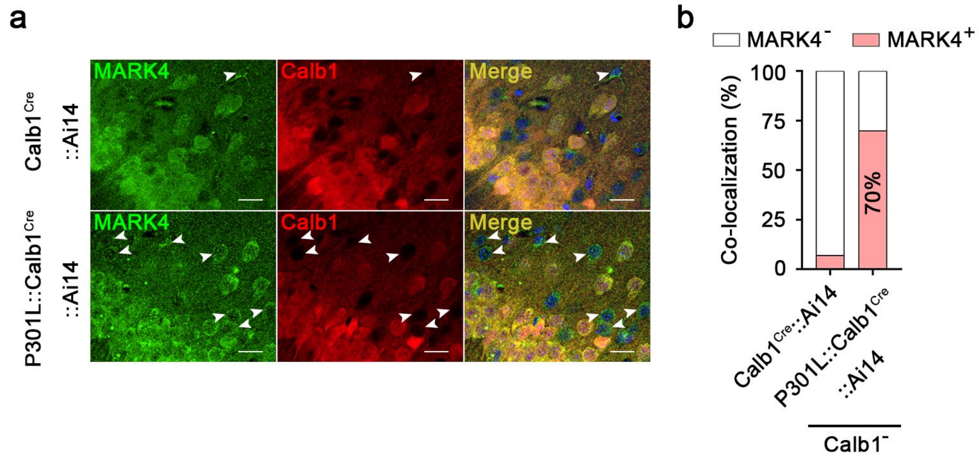

Figure S14. MARK4 is significantly increased in Calb1- neurons in the dvCA1 of P301L::Calb1-IRES2-Cre-D::Ai14 mice. (**a**) Representative images showed MARK4 immunostaining in P301L::Calb1-IRES2-Cre-D::Ai14 mice. Arrow heads indicated cells that were MARK4+ and Calb1-. Scale bar = 20 μm. (**b**) Quantitative analysis showed that 70% of Calb1- neurons in the dvCA1 of P301L::Calb1-IRES2-Cre-D::Ai14 mice exhibited MARK4+ signals.

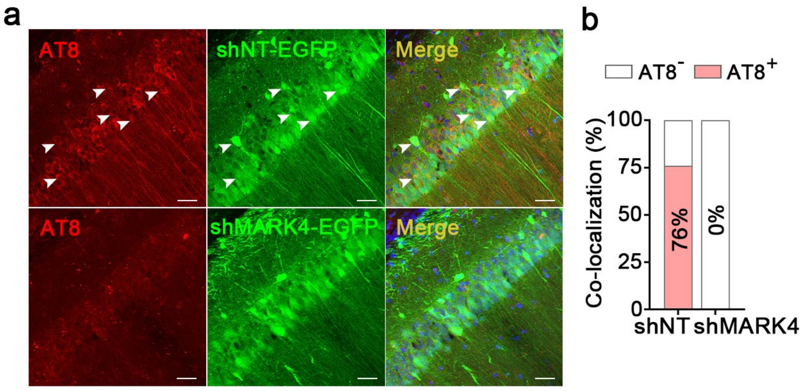

Figure S15. Knocking down MARK4 reduces accumulation of hyperphosphorylated Tau in the dvCA1 of P301L mice. (**a**) Representative images showing that knocking down MARK4 effectively reduces phosphorylated tau in the dvCA1 of P301L mice. Arrows indicated co-labeled cells. (**b**) Quantitative analysis showed that knockdown of MARK4 reduced 76% $EGFP^+$ and $AT8^+$ signals to 0% in P301L mice. Scale bar = 50 μm.

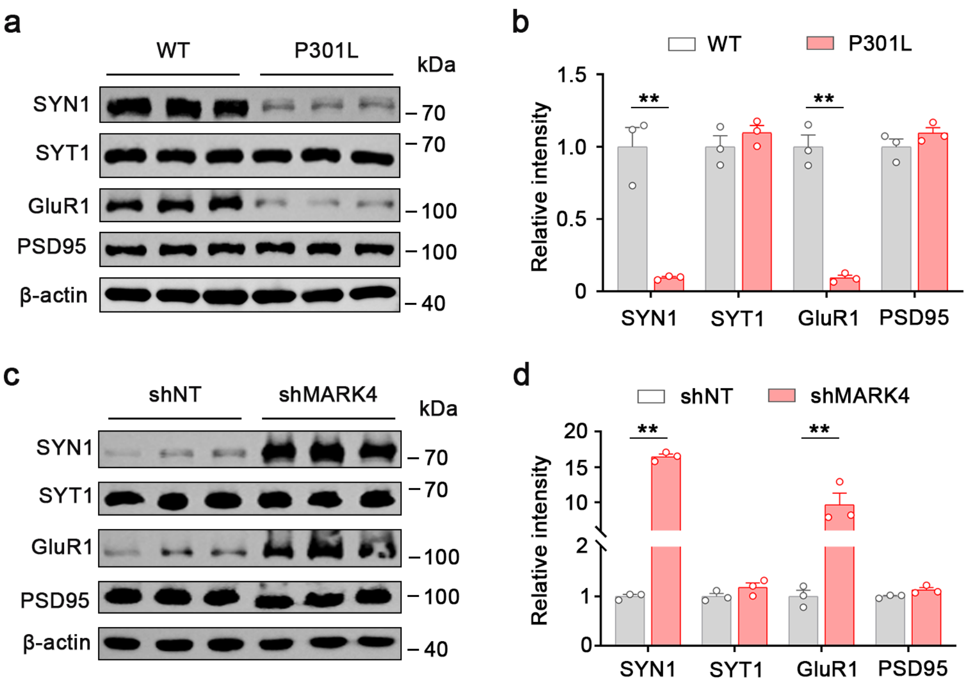

Figure S16. Knocking down MARK4 in the dvCA1 attenuates tau-induced synaptic protein loss in P301L mice. (**a, c**) Western blotting showed the protein levels of SYN1, SYT1, GluR1 and PSD95 in the dvCA1 of 3-month-old P301L mice and controls. (**b, d**) Quantitative analysis revealed that the protein levels of SYN1 and GluR1 were significantly decreased in the P301L group compared to the WT group but increased notably following MARK4 knockdown. No significant differences in SYT1 and PSD95 were detected between the groups. Two-tailed unpaired *t*-test, SYN1: $t = 6.740$, $df = 4$, $P = 0.0025$ (**b**), $t = 41.31$, $df = 4$, $P < 0.0001$ (**d**); SYT1: $t = 1.060$, $df = 4$, $P = 0.3490$ (**b**), $t = 1.652$, $df = 4$, $P = 0.1739$ (**d**); GluR1: $t = 10.75$, $df = 4$, $P = 0.0004$ (**b**), $t = 5.315$, $df = 4$, $P = 0.006$ (**d**); PSD95: $t = 1.427$, $df = 4$, $P = 0.2267$ (**b**), $t = 2.564$, $df = 4$, $P = 0.0624$ (**d**). $n = 3$ mice per group. Data were presented as mean ± SEM., $^{**}P < 0.01$.

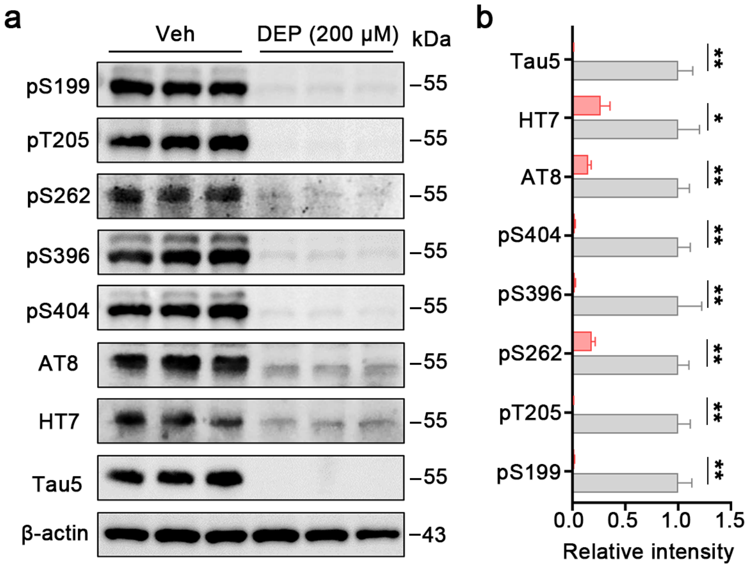

Figure S17. DEPTAC reduces pathological Tau in primary neurons instantly transfected with hTau. (**a, b**) DEPTAC at 200 μM dramatically reduced total Tau (Tau5, HT7) and phosphorylated Tau (pS199, pT205, pS262, pS396, pS404 and AT8) in primary neurons instantly transfected with hTau. Two-tailed unpaired *t*-test, pS199: $t = 7.457$, $df = 4$, $P = 0.0017$; pS205: $t = 8.393$, $df = 4$, $P = 0.0011$; pS262: $t = 7.463$, $df = 4$, $P = 0.0017$; pS396: $t = 4.308$, $df = 4$, $P = 0.0126$; pS404: $t = 8.232$, $df = 4$, $P = 0.0012$; AT8: $t = 7.601$, $df = 4$, $P = 0.0016$; HT7: $t = 3.295$, $df = 4$, $P = 0.0301$; Tau5: $t = 6.971$, $df = 4$, $P = 0.0022$ (**b**). Data were presented as mean ± SEM., $^{*}P < 0.05$, $^{**}P < 0.01$.

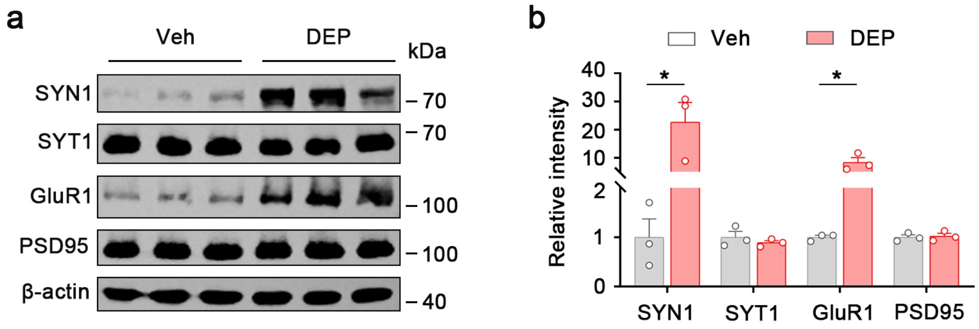

Figure S18. DEPTAC treatment increased synaptic proteins in P301L mice. (**a**) Western blotting showed the protein levels of SYN1, SYT1, GluR1 and PSD95 in the dvCA1 of 3-month-old P301L mice with or without DEPTAC (DEP) administration. (**b**) Quantitative analysis revealed that the protein levels of SYN1 and GluR1 were significantly increased in DEP group compared to Vehicle (Veh) group. No significant differences in SYT1 and PSD95 were detected between the groups. Two-tailed unpaired *t*-test, SYN1: $t = 3.117$, $df = 4$, $P = 0.0356$; SYT1: $t = 0.8895$, $df = 4$, $P = 0.4240$; GluR1: $t = 4.241$, $df = 4$, $P = 0.0133$; PSD95: $t = 0.2790$, $df = 4$, $P = 0.7941$. $n = 3$ mice per group. Data were presented as mean ± SEM., $^*P < 0.05$.

Table S1 The information of the human subjects

CBB code	Sex	Age (years)	Braak	APOE	Post-mortem delay (MIN)	CSF PH	BW (g)	Diagnosis	Specific
2019CBB041	M	86	0	E3	270	6.18	1104	Non-demented control	Anterior hippocampus
	M	86	0	E3	270	6.18	1104	Non-demented control	Posterior hippocampus
2022CBB031	M	86	3	E3	215	6.59	1233	Alzheimer's disease	Anterior hippocampus
	M	86	3	E3	215	6.59	1233	Alzheimer's disease	Posterior hippocampus

Table S2 Virus strains and their applications

Virus strains	Source	Identifier	For experiment
rAAV-CaMKIIa-DO-Tau(human)-mCherry	Brain Case	Cat# BC-0340	Behavioral test or in vitro Ca^{2+} imaging in brain slices
rAAV-CaMKIIa-DO-mCherry	Brain Case	Cat# BC-0349	Behavioral test or in vitro Ca^{2+} imaging in brain slices
rAAV-CaMKIIa-DO-hM4D(Gi)-mCherry	Brain Case	Cat# BC-0348	Behavioral test
pAAV-hSyn-shMARK4-eGFP	Brain Case	Cat# BC-1146	Behavioral test or Western blotting
pAAV-hSyn-shNT-eGFP	Brain Case	Cat# BC-0186	Behavioral test or Western blotting
rAAV-EF1a-DO-GCaMPf-eGFP	OBio	N/A	in vivo Ca^{2+} recording or in vitro Ca^{2+} imaging in brain slices
rAAV-EF1a-DO-eGFP	OBio	N/A	in vivo Ca^{2+} recording
pLenti-hSyn-MAPT-eGFP	OBio	N/A	Cell transfection

Table S3 Antibody list

Antibodies	Host	Source	Cat#	Dilution
Calbindin	Rabbit	Abcam	ab108404	1:200 (IF)
MARK4	Rabbit	CST	4834	1:200 (IF); 1:500 (WB)
MARK4	Rabbit	Proteintech	20174-1-AP	1:150 (IF)
AT8	Mouse	Thermo	MN1020	1:200 (IF); 1:1000 (WB)
Iba1	Rabbit	FUJIFILM Wako Pure Chemical Corporation	019-19741	1:400 (IF)
NeuN	Mouse	CST	94403	1:200 (IF)
pS199	Rabbit	Invitrogen	44-734G	1:1000 (WB)
pT205	Rabbit	SAB	11108	1:1000 (WB)
pS262	Rabbit	SAB	11111	1:1000 (WB)
pS356	Rabbit	Invitrogen	44-751G	1:1000 (WB)
pS396	Rabbit	SAB	11102	1:1000 (WB)
pS404	Rabbit	SAB	11112	1:1000 (WB)
HT7	Mouse	Thermo	MN1000	1:1000 (WB)
Tau5	Mouse	abcam	ab80579	1:1000 (WB)
β-actin	Rabbit	abclonal	AC026	1:1000 (WB)
SYN1	Rabbit	abclonal	A17362	1:1000(WB)
PSD95	Rabbit	abclonal	A0131	1:1000(WB)
GluR1	Rabbit	abclonal	A11643	1:1000(WB)
SYT1	Rabbit	abclonal	A0992	1:1000(WB)
HRP-conjugated goat anti-rabbit IgG	Goat	Beyotime	A0208	1:3000 (WB)
HRP-conjugated goat anti-mouse IgG	Goat	Beyotime	A0216	1:3000 (WB)
Alexa Fluor 488 donkey-anti-rabbit IgG (H+L)	Donkey	Invitrogen	A21206	1:500 (IF)
Alexa Fluor 488 donkey-anti-mouse IgG (H+L)	Donkey	Invitrogen	A21202	1:500 (IF)
Alexa Fluor 546 donkey-anti-rabbit IgG (H+L)	Donkey	Invitrogen	A10040	1:500 (IF)
Alexa Fluor 546 donkey-anti-mouse IgG (H+L)	Donkey	Invitrogen	A10036	1:500 (IF)
