# Supplementary figures and images for: Improving vulnerable Calbindin1− neurons in the ventral hippocampus rescues tau-induced impairment of episodic memory
Source: Transl Neurodegener. 2025 Mar 4;14:12. doi: 10.1186/s40035-025-00473-w (PMC11877784; doi:10.1186/s40035-025-00473-w)

**Figure 3b**


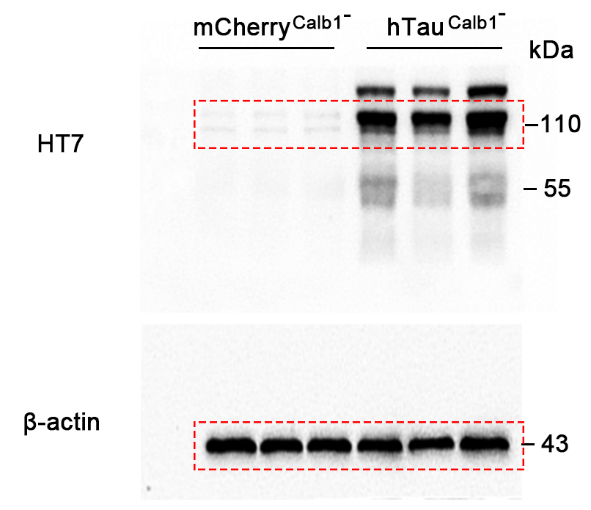


**Figure 6d**


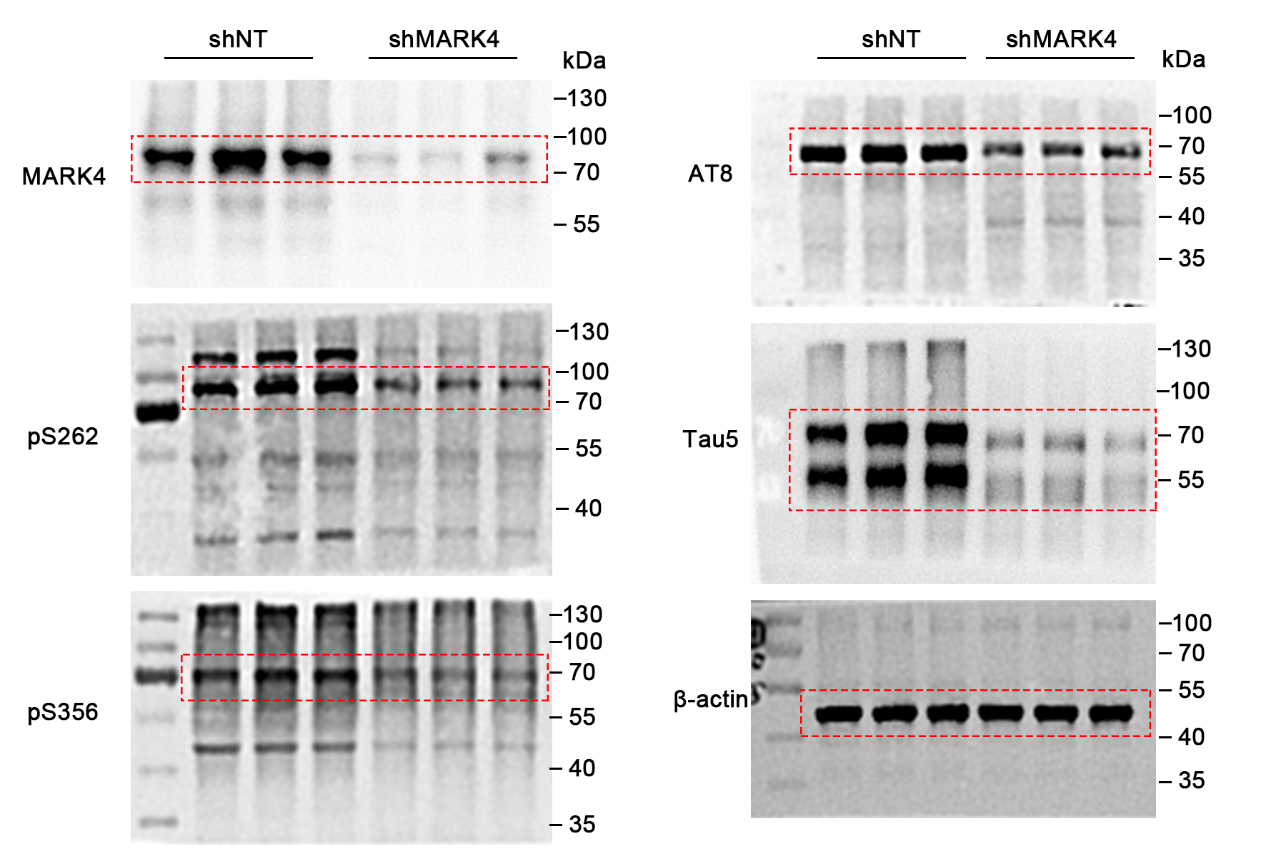


**Figure 7b**


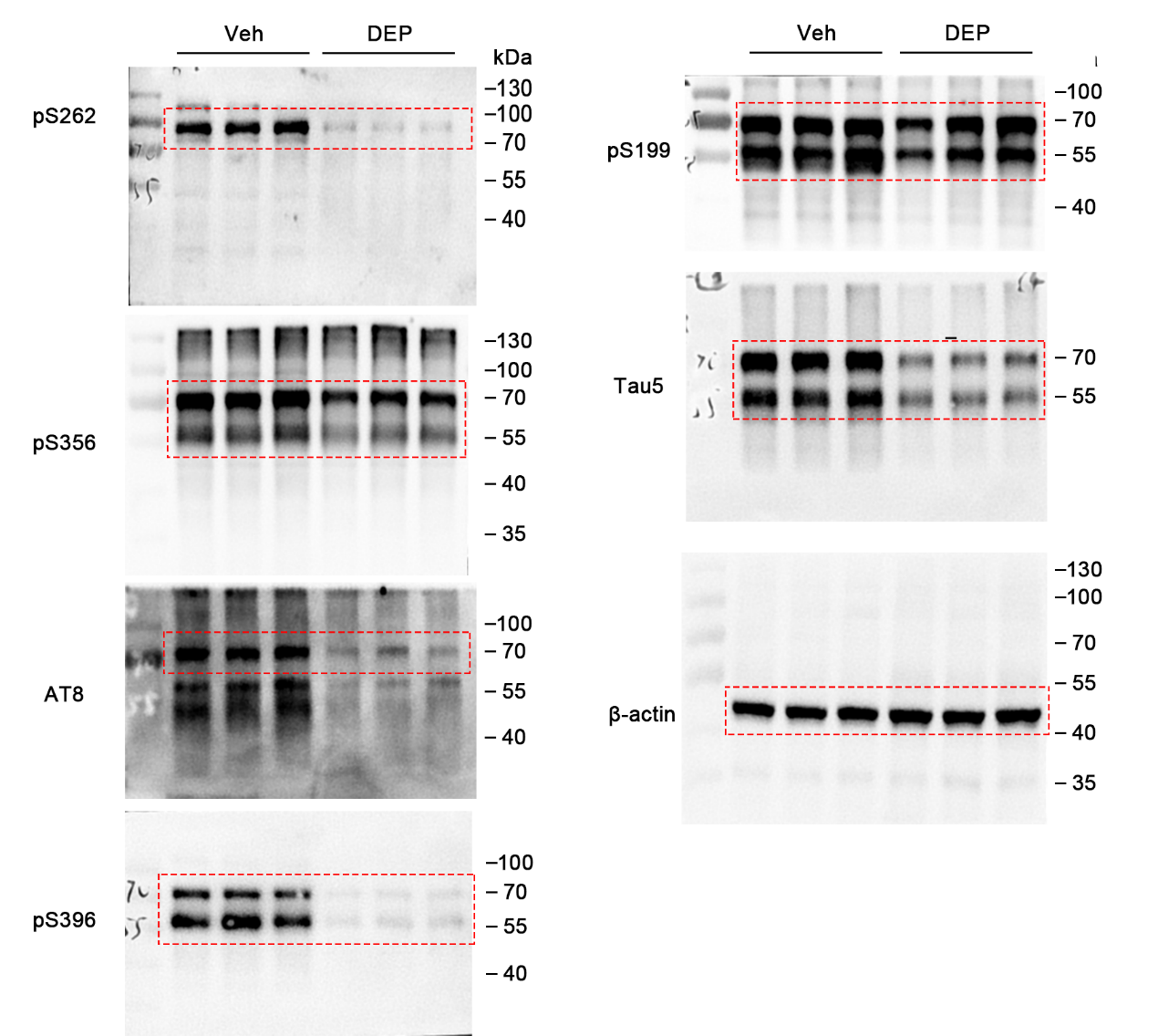


**Figure S4a**


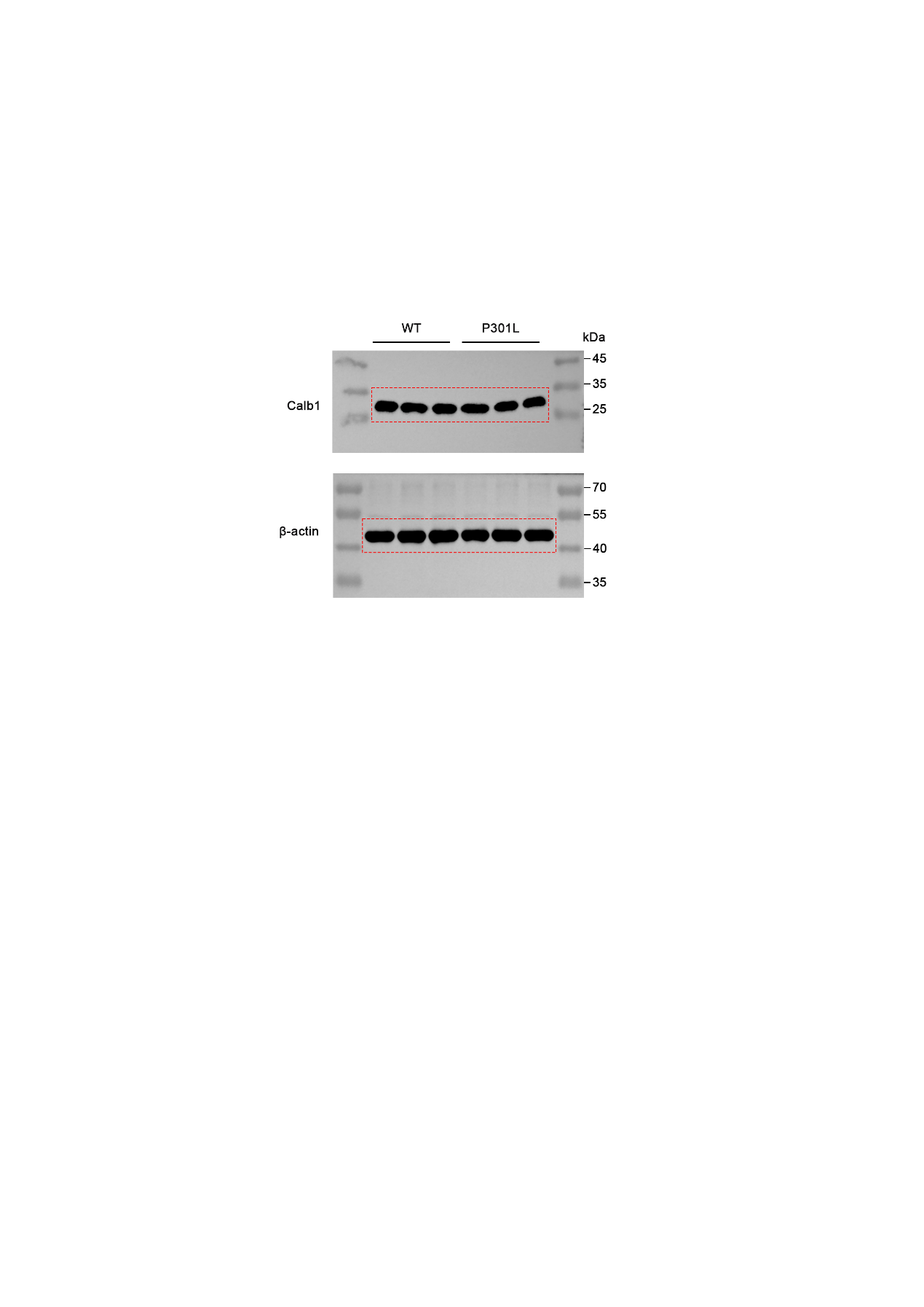


**Figure S16a Figure S16c**


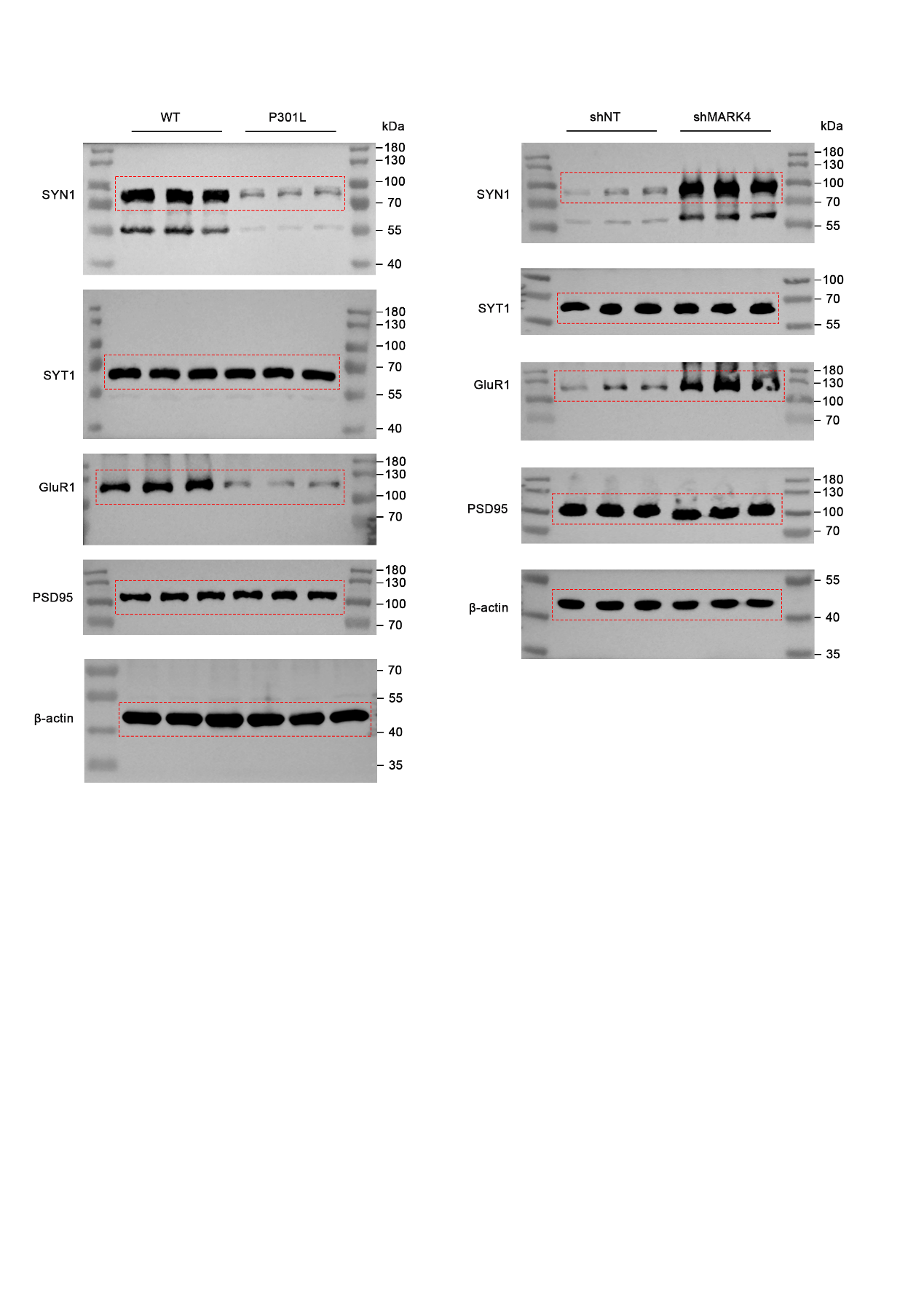


**Figure S17a**


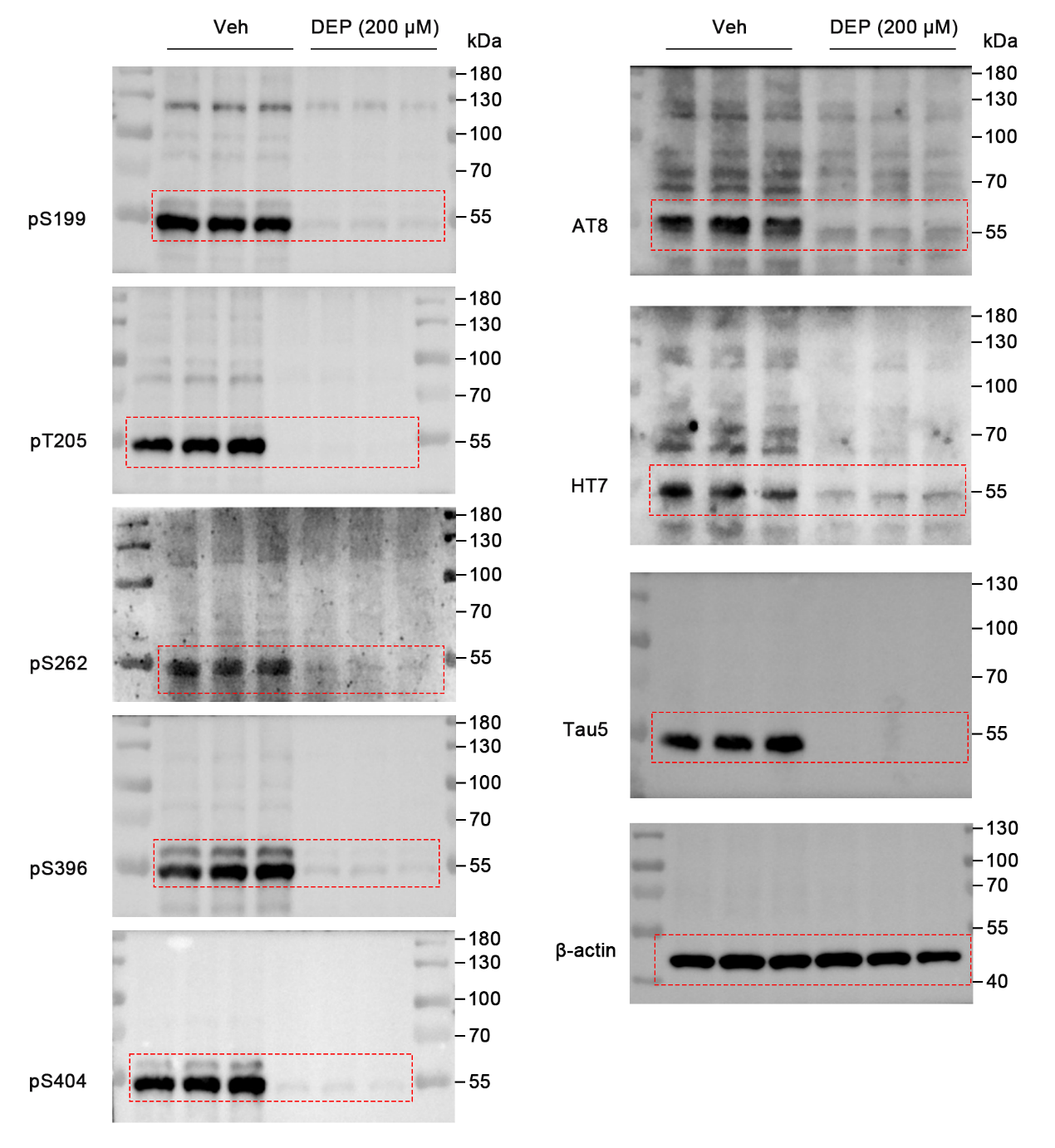


**Figure S18a**


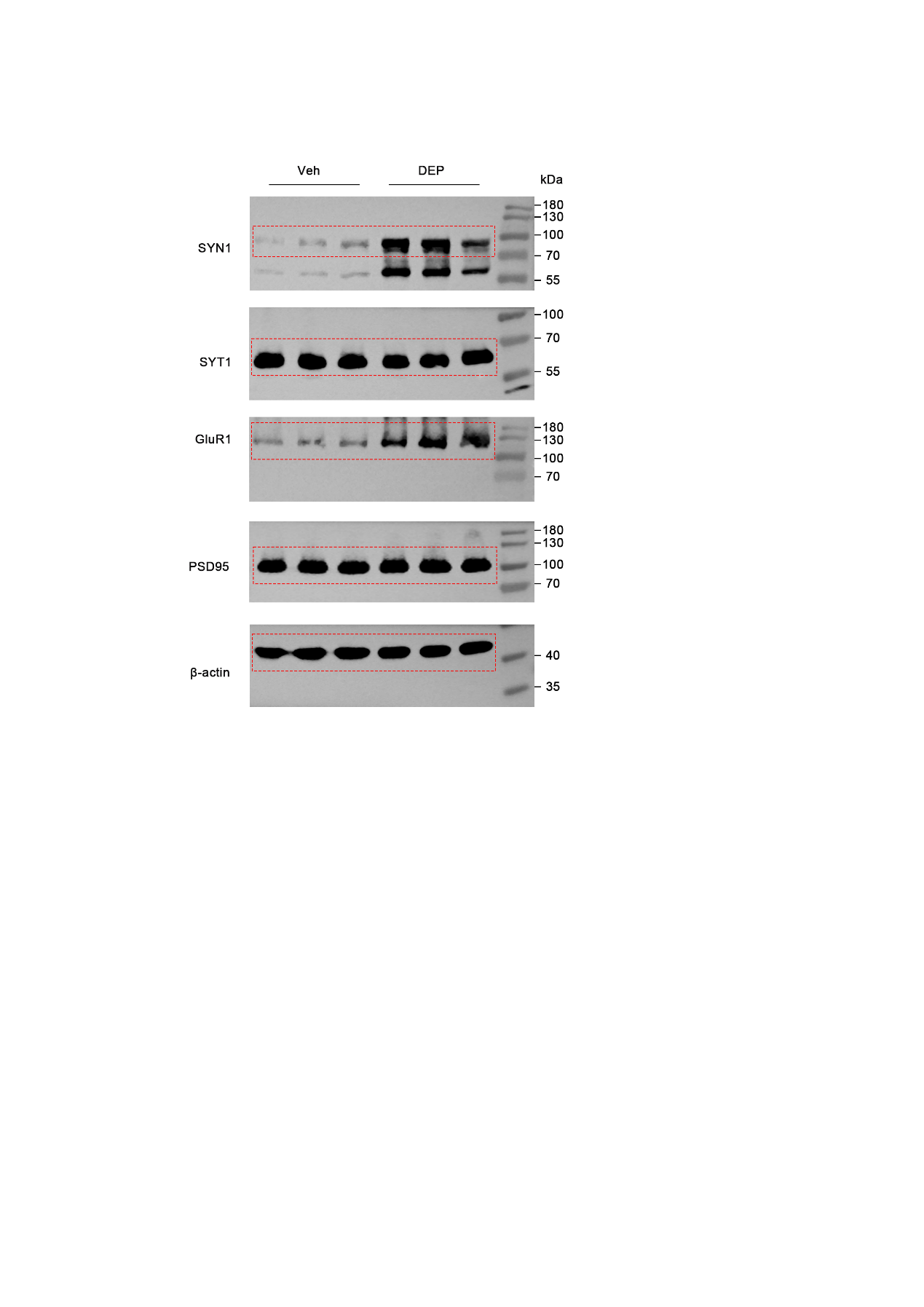

Supplement: Supplementary file 2 — Additional file 2. Full Western blots images. [file 40035_2025_473_MOESM2_ESM.docx]
